# Integrated single-cell analysis characterizes malignant epithelial programs and context-dependent immune associations of MP7/SERINC2 in colorectal cancer

**DOI:** 10.3389/fimmu.2026.1918381

**Published:** 2026-09-03

**Authors:** Wei Ying, Yuhan Zhang, Shirong Wu, Rui Wu, Xiaoxia Li

**Affiliations:** 1 Department of Radiotherapy, Sichuan Clinical Research Center for Cancer, Sichuan Cancer Hospital and Institute, Sichuan Cancer Center, University of Electronic Science and Technology of China, Chengdu, China; 2 Department of Gastric Surgery, Sichuan Clinical Research Center for Cancer, Sichuan Cancer Hospital and Institute, Sichuan Cancer Center, University of Electronic Science and Technology of China, Chengdu, China; 3 Department of Medical Oncology, Sichuan Clinical Research Center for Cancer, Sichuan Cancer Hospital and Institute, Sichuan Cancer Center, Affiliated Cancer Hospital of University of Electronic Science and Technology of China, Chengdu, China

**Keywords:** colorectal cancer, immune checkpoint blockade, malignant epithelial cells, metaprogram, SERINC2, single-cell RNA sequencing, tumor immune microenvironment

## Abstract

**Introduction:**

Colorectal cancer (CRC) comprises heterogeneous malignant epithelial states, but it remains unclear which transcriptional programs recur across patients and how these programs relate to the immune context.

**Methods:**

We integrated five public single-cell RNA-sequencing datasets comprising 425,402 cells from 119 patients using scVI, defined malignant epithelial cells with inferCNV, and characterized their states and transcriptional programs using cNMF, pySCENIC, and CytoTRACE2. Program recurrence was assessed by rerunning cNMF separately for individual patients. Metaprogram associations were subsequently evaluated in bulk cohorts, CRC cell lines, spatial transcriptomic datasets, and an immune-checkpoint inhibitor (ICI) cohort.

**Results:**

We identified five malignant epithelial states and eight metaprograms. MP7, a secretory/goblet-lineage program, was independently recovered in 9 of 26 evaluable patients across three datasets, with reciprocal-best matches in five. Although its top-100 gene profile closely resembled that of normal goblet cells (Spearman r = 0.834), 92.2–94.2% of MP7-dominant cells were retained as malignant under stricter classification criteria. Bulk MP7 scores were sensitive to epithelial composition, and an exploratory 17-gene survival score derived from MP7 showed limited discrimination in nested cross-validation and external cohorts. SERINC2 was prioritized from this gene set for functional testing. SERINC2-targeting shRNAs increased wound closure and Transwell invasion in SW480 and RKO cells, with RNA-level knockdown confirmed in SW480 cells. MP7 and SERINC2 showed context-dependent associations with immune features at the patient and spatial levels, but neither distinguished response in the examined ICI cohort.

**Discussion:**

MP7 represents a secretory/goblet-lineage program that recurs in a subset of CRC tumors rather than a broadly shared malignant state. The findings also identify SERINC2 as a candidate regulator of CRC cell motility and invasion, although its mechanism and clinical relevance require further validation.

## 1 Introduction

Colorectal cancer (CRC) is among the most frequently diagnosed malignancies worldwide and remains a major cause of cancer-related mortality (1). Its clinical behavior is heterogeneous because tumor evolution is shaped by anatomical location, genomic and epigenomic alterations, differentiation state, treatment exposure, and the composition of the tumor microenvironment (2). Immune contexture has established prognostic value in localized colon cancer (3), and immune-checkpoint blockade can induce durable responses in mismatch-repair-deficient or microsatellite-instability-high tumors (4). Nevertheless, most CRCs are mismatch-repair proficient, and the cellular programs that coexist with or organize different immune ecosystems remain insufficiently defined.

Bulk transcriptomic studies established the consensus molecular subtype framework, which partitions CRC into immune, canonical, metabolic, and mesenchymal classes (5). Single-cell analyses subsequently refined this framework by identifying two intrinsic malignant epithelial states, iCMS2 and iCMS3, and demonstrated that some bulk classifications primarily reflect tumor-cell-intrinsic programs whereas others are strongly influenced by stromal or immune admixture (6). This distinction is biologically important because malignant epithelial lineage, differentiation, stress, and secretory programs can alter antigen presentation, chemokine production, metabolic competition, and the physical organization of the immune microenvironment.

Several single-cell studies have delineated CRC heterogeneity at high resolution. Paired tumor-mucosa profiling revealed lineage-dependent malignant programs linked to immune landscapes (7); other studies identified MAPK-dependent epithelial trajectories (8), spatially organized immune-malignant hubs (9), and cell states that refine clinical stratification (10). Single-cell chromatin accessibility further demonstrated that CRC molecular subtypes are supported by distinct regulatory elements and transcription-factor activities (11). However, discrete clustering alone cannot fully capture transcriptional programs that recur across patients, coexist within the same cell, or vary continuously across malignant states. Moreover, regulon-level information remains incompletely integrated with recurrent malignant-cell programs.

**Figure 1:**
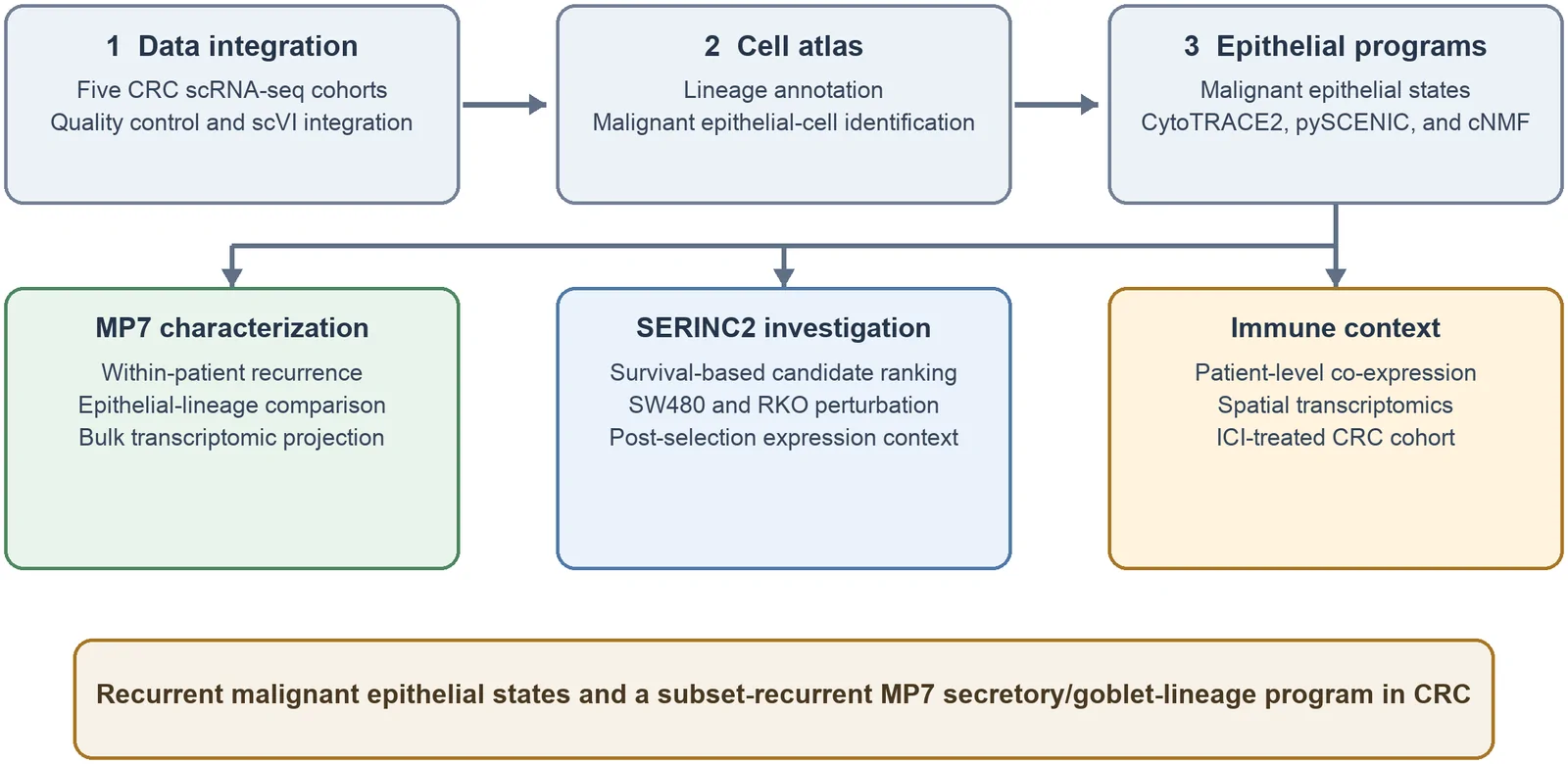
Study design and analytical workflow. Five CRC scRNA-seq cohorts were integrated to define cell types, putative malignant epithelial cells, cell states, and expression metaprograms. MP7 and SERINC2 were subsequently examined using bulk transcriptomic cohorts, cell-line perturbation, and spatial and immune-context analyses.

Consensus non-negative matrix factorization (cNMF) can resolve expression programs that coexist within individual cells (12), whereas pySCENIC infers transcription-factor-centered regulons from coexpression and motif enrichment (13, 14). D-SPIN estimates condition-aware statistical couplings in single-cell data (15). Here, we integrated five CRC scRNA-seq cohorts to identify malignant epithelial states and their associated expression programs. CytoTRACE2 and pySCENIC characterized state-associated features, whereas cNMF identified programs whose recurrence was evaluated within individual patients. This patient-level analysis extended previous epithelial-state classifications by distinguishing widely recovered programs from programs found in only a subset of tumors. The MP7-derived candidate set was then used to prioritize SERINC2 for functional study. We further examined MP7 and SERINC2 using bulk cohorts, cell-line assays, D-SPIN analysis, spatial data, and an ICI-treated cohort (**Figure 1**).

## 2 Materials and methods

### 2.1 Study design and data sources

This study integrated five publicly available human CRC scRNA-seq datasets: GSE132465 and GSE144735 from Lee et al. (7), GSE166555 from Uhlitz et al. (8), GSE178341 from Pelka et al. (9), and GSE200997 from Khaliq et al. (10). Count matrices and sample metadata were harmonized while retaining patient, sample, tissue, and dataset identifiers. After quality control, the combined dataset comprised 425,402 cells, 16,126 genes, 119 patients, and 199 tumor, adjacent, or normal colorectal samples.

TCGA-COAD RNA-seq and clinical data were used for metaprogram scoring, survival analysis, and candidate-gene selection (2). GSE12945, GSE39084, GSE39582, and GSE17536 were used to assess the exploratory MP7-derived 17-gene score (16–19); GSE39582 and GSE17536 were also used to assess SERINC2 survival associations (18, 19). Samples lacking the required expression or overall-survival data were excluded.

### 2.2 Single-cell quality control, doublet removal, scVI integration, and cell-type annotation

Cells were retained if they contained at least 500 UMI counts, expressed at least 200 genes, and had a mitochondrial-transcript fraction of no more than 15%. Genes detected in fewer than 10 cells were removed. Doublets were evaluated within each sample using Scrublet with an expected doublet rate of 0.08 (20) and SOLO with 3,000 highly variable genes and 100 training epochs (21); cells classified as doublets by the consensus procedure were excluded.

The datasets were integrated using scVI implemented in scvi-tools v1.4.3 (22), with 4,000 highly variable genes and sample_uid as the batch covariate. The model used a 30-dimensional latent space, two hidden layers, 128 hidden units, a batch size of 2,048, and 120 training epochs. The latent representation was used for neighbor-graph construction, UMAP visualization, and Leiden clustering at resolution 0.6. Sensitivity to the batch covariate was evaluated using matched scVI models with sample_uid, dataset, or no batch covariate (**Supplementary Methods** and **Supplementary Figure 1**). Cell types were annotated using canonical lineage markers, cluster-specific markers, and UMAP topology. Epithelial, T-cell, B-cell/plasma-cell, myeloid, and stromal populations were identified using established marker combinations, including EPCAM and keratins, CD3D, CD4, CD8A, MS4A1, CD79A, JCHAIN, LYZ, C1QA, COL1A1, PECAM1, and RGS5.

### 2.3 Expression-inferred copy-number variation and malignant-cell identification

Large-scale copy-number-associated expression changes were inferred using an inferCNV-based workflow, with immune cells as the reference and epithelial-lineage cells as the query population (23). Epithelial cells with an inferCNV-derived score > 0.016629 were classified as putative malignant cells, yielding 22,848 cells for subsequent state and metaprogram analyses.

Malignant-cell assignments were further assessed using patient-calibrated CNV burden and scMalignantFinder v1.2.0 (24). CNV burden was compared with normal epithelial cells from the same patient or, when unavailable, from the same dataset. scMalignantFinder probabilities were similarly calibrated against normal epithelial cells within each dataset. Detailed calibration and sensitivity-analysis procedures are provided in the **Supplementary Methods** and **Supplementary Figure 2**.

### 2.4 Differential expression and pathway enrichment

Genes upregulated in putative malignant relative to nonmalignant epithelial cells were analyzed for Gene Ontology Biological Process enrichment. For preranked gene set enrichment analysis (GSEA), genes were ordered by the signed differential-expression statistic and tested against Gene Ontology Biological Process 2023 and MSigDB Hallmark gene sets (25, 26). Results were reported as normalized enrichment scores (NES), nominal P values, and false-discovery-rate (FDR) q values. Statistical significance was defined by FDR< 0.05; nominal P values were reported for completeness but were not used alone to determine significance. A positive NES indicated enrichment toward genes upregulated in putative malignant cells.

### 2.5 Malignant epithelial reclustering and CytoTRACE2

The 22,848 putative malignant epithelial cells were extracted from the raw-count matrix and reclustered using a separate scVI model with 3,000 highly variable genes, sample_uid as the batch covariate, and a 30-dimensional latent space. The latent representation was used for UMAP visualization and Leiden clustering at resolution 0.4. Raw counts were normalized to 10,000 counts per cell and log transformed, and cluster-specific markers were identified using the Wilcoxon rank-sum test. The five clusters were annotated as TUBA1B+, GPR160+, TM4SF1+, SERPINA1+, and ARGLU1+ malignant epithelial states.

CytoTRACE2 v1.1.0 was applied to the raw UMI count matrix using the human model (27) to calculate continuous potency scores, relative-order scores, and discrete potency categories. State recurrence and cell-cycle sensitivity were evaluated as described in the **Supplementary Methods** and **Supplementary Figure 3**.

### 2.6 pySCENIC regulon analysis

Transcription-factor-centered regulons were inferred from the raw UMI count matrix of the 22,848 putative malignant epithelial cells using pySCENIC v0.12.1 (13, 14). Transcription factor-target coexpression relationships were identified with GRNBoost2 implemented in Arboreto v0.1.6, using the hg38 human transcription-factor list. Coexpression modules were pruned by cis-regulatory motif enrichment using the hg38 10-kb-upstream/10-kb-downstream and 500-bp-upstream/100-bp-downstream cisTarget v10 ranking databases together with the motifs-v10nr HGNC annotation database. Dropout masking was enabled during motif pruning.

The activity of each retained regulon was quantified in individual cells using AUCell. Regulon specificity scores were calculated from the cell-level AUCell matrix to identify regulons preferentially active in each malignant epithelial state. The five regulons with the highest specificity scores for each state were selected for visualization. Regulon size was defined as the number of target genes retained after motif-based pruning. Cross-dataset and within-patient recurrence analyses are described in the **Supplementary Methods**.

### 2.7 cNMF metaprogram discovery and functional annotation

Consensus non-negative matrix factorization was applied to the raw-count matrix of 22,848 putative malignant epithelial cells using cNMF v1.7.1 (12). The analysis used 3,000 highly variable genes and evaluated candidate ranks from K = 4 to K = 12. Each rank was run 80 times, with a maximum of 1,000 iterations per run. K = 8 was selected based on consensus stability and reconstruction error, yielding eight metaprograms designated MP1-MP8.

Genes within each metaprogram were ranked by their cNMF gene-spectrum scores. Cell-level usage scores were used to determine the dominant metaprogram and its association with the five malignant epithelial states. Functional annotation was performed by correlating metaprogram usage with predefined biological signature scores and CytoTRACE2 scores using Spearman correlation. Patient-level recurrence, epithelial-lineage comparisons, and single-cell-to-bulk transfer analyses are described in the **Supplementary Methods**.

### 2.8 TCGA-COAD metaprogram scoring and survival analysis

TCGA-COAD primary-tumor TPM expression and clinical follow-up data were analyzed. MP1-MP8 scores were calculated from the 200 highest-ranking genes in each cNMF program using single-sample gene set enrichment analysis implemented in GSVA v2.0.7 (28). Expression values were transformed as log2(TPM + 1), and ssGSEA scores were normalized using ssgseaParam. The MP7 top-100 score was calculated using the same procedure for covariate-adjusted analyses.

Immune and stromal cell abundances were estimated using MCP-counter v1.2.0 with HUGO gene symbols (29). Consensus molecular subtypes were assigned using CMScaller v0.99.2 in RNA-seq mode with 1,000 permutations and an FDR threshold of 0.05 (30).

Survival analyses included 389 primary tumors with valid overall-survival data, including 95 deaths. Cox proportional hazards models were fitted using survival v3.8-3, with continuous scores standardized before analysis. Multivariable models adjusted for age, sex, and pathological stage. ABSOLUTE tumor purity, mucinous histology, MSI status, tumor location, or CMS classification was included in separate models. Complete cases were used for each model, and proportional-hazards assumptions were evaluated using Schoenfeld residuals. Kaplan-Meier analyses divided patients at the median ssGSEA score, and survival distributions were compared using log-rank tests.

### 2.9 Construction and performance assessment of an exploratory MP7-derived 17-gene survival score

The top 300 MP7-ranked genes were evaluated in 389 TCGA-COAD tumors with overall-survival data. Expression was transformed as log2(TPM + 1), and 298 genes were detected. Thirty-two genes associated with overall survival in univariable Cox analyses (*P<* 0.05) were entered into LASSO-Cox regression using glmnet .v4.1-8 (α=1, 10-fold cross-validation, lambda.min, lambda.min.ratio=0.01, and a maximum of 10^6^ iterations) (31). Seventeen genes were retained: HSPA1A, SERPINA1, ATOH1, UGT2B7, BCL10, ASRGL1, ITLN1, CEACAM5, SLC39A8, AFAP1-AS1, KREMEN2, KLK12, ADAM8, C3orf70, FAM177B, SLC9A3R2, and SERINC2. These genes were fitted in a multivariable Cox model, with *β*_i_ denoting the fitted coefficient and E_i_ the expression of gene i. The risk score was calculated as follows:


$$Risk\;score=\sum \beta_{i}E_{i}$$

Patients were divided at the median risk score for Kaplan-Meier analysis and log-rank testing. The association between risk group and overall survival was further evaluated in a Cox model adjusted for age, sex, and simplified pathologic stage. Time-dependent AUCs at 1, 3, and 5 years were calculated using timeROC v0.4. Internal performance was estimated using 20 repeats of five-fold nested cross-validation, with univariable screening and inner five-fold LASSO-Cox fitting repeated within each training partition. Model performance was summarized using out-of-fold Harrell C-index. In a separate repeated five-fold analysis of 349 complete cases, a 17-gene Cox model, a clinical model including age, sex, and stage, and a combined model were fitted in each training fold and evaluated by held-out C-index, calibration slope, and inverse-probability-of-censoring-weighted Brier scores at 3 and 5 years. Cox models were fitted using survival v3.8-3.

GSE12945 and GSE39084 were used for Kaplan-Meier assessment of the 17-gene score using the TCGA-derived coefficients without refitting (16, 17). Additional evaluation was performed in GSE39582 and GSE17536 (18, 19). Fifteen model genes were available; UGT2B7 and FAM177B were absent. The TCGA-derived coefficients were applied without re-estimation, and the resulting continuous scores were tested using unadjusted and age-, sex-, and stage-adjusted Cox models. Discrimination was evaluated by comparing the C-indices of the gene-score, clinical, and combined models.

### 2.10 Candidate-gene ranking and external SERINC2 analysis

The 17 genes retained by LASSO-Cox were further ranked in TCGA-COAD using three survival-learning methods. Random survival forests ranked genes by permutation variable importance (32), XGBoost-Cox ranked genes by mean absolute SHAP values (33, 34), and CoxBoost ranked genes by absolute penalized coefficients (35). The analyses used randomForestSRC v3.5.1, xgboost v3.2.1.1, and CoxBoost v1.5.1, respectively. Ranking stability was assessed using 100 random subsamples containing 80% of patients. The three highest-ranked genes from each method were recorded in each subsample. Normalized method-specific ranks and mean selection frequencies were combined into a consensus score, and the three genes with the highest scores were selected for further analysis.

SERINC2 expression was subsequently evaluated in GSE39582 and GSE17536 (18, 19). Associations with overall survival were tested using univariable and age-, sex-, and stage-adjusted Cox models. Cohort-specific estimates were combined using fixed-effect meta-analysis.

### 2.11 Cell culture, SERINC2-targeting shRNA transduction, and RT-qPCR validation

SW480 and RKO human CRC cells obtained from the Chinese Academy of Sciences Type Culture Collection were cultured in DMEM (Gibco, USA) supplemented with 10% fetal bovine serum and 1% penicillin/streptomycin at 37 °C in a humidified atmosphere containing 5% CO_2_. For lentiviral shRNA transduction, the SERINC2-targeting sequences SH-A (5′-GTGCTGGTGTCCATCATTATG-3′) and SH-B (5′-CGCCTCATCTTCACGTTCTTC-3′) were cloned into the pLKO.1-TRC vector and verified by Sanger sequencing. Lentivirus was generated by co-transfecting 293T cells with the shRNA construct, psPAX2, and pMD2.G using Lipofectamine 3000 (Invitrogen, USA). Viral supernatants were collected after 72 hours, filtered through a 0.45-µm membrane, and used to infect logarithmically growing SW480 and RKO cells in the presence of polybrene (2 µg/mL). Puromycin selection (4 µg/mL) was initiated after 48 hours and continued for 5 days. Total RNA was extracted from SW480 cells using TRIzol reagent and reverse-transcribed with the ReverTra Ace qPCR RT Kit (TOYOBO, FSQ-101). Quantitative PCR was performed on a LightCycler 96 system (Roche) using 2× SYBR Green Fast qPCR Mix (ABclonal, RK21203) according to the manufacturer’s instructions. The primer sequences were as follows: SERINC2, forward 5′-GGTGCCTTCTACATTCCTGACG-3′ and reverse 5′-TGGTTCCAGGAGTGCGCAAAGT-3′; ACTB, forward 5′-CATGTACGTTGCTATCCAGGC-3′ and reverse 5′-CTCCTTAATGTCACGCACGAT-3′. ACTB was used as the reference gene, and relative SERINC2 expression was calculated using the 2^−ΔΔCt^ method with SH-NC as the calibrator. Results are presented as the mean ± SD of three independent experiments, and knockdown efficiency was calculated relative to SH-NC.

### 2.12 Wound-healing and Transwell invasion assays

For wound-healing assays, stable cells were seeded in six-well plates and grown to approximately 95% confluence. A linear scratch was generated with a sterile 200-µL pipette tip, detached cells were removed with phosphate-buffered saline, and fresh medium was added. Matched fields were imaged at 0, 24, and 48 hours. For invasion assays, cells at 2.0×10^5^ cells/mL in serum-free medium were placed in the upper chamber of 8-µm-pore Transwell inserts pre-coated with Matrigel, while medium containing 10% fetal bovine serum was added to the lower chamber. After 24 hours, invaded cells were fixed, stained with crystal violet, imaged, and counted. For the RKO assays, 24-hour wound closure was calculated as 100 minus the remaining-wound percentage, and invaded-cell counts were recorded at 24 hours. Each group contained three measurements. Replicate-blocked linear models with Dunnett adjustment compared SH-NC, SH-A, and SH-B with NC.

### 2.13 D-SPIN analysis of the SERINC2 expression context

D-SPIN v1.5.2 was applied to library-size-normalized, log-transformed epithelial expression data (15). SERINC2 was retained as the index gene, and the remaining features were selected from dataset-aware highly variable genes using Scanpy v1.12.1 (36). Conditions were defined by sample_uid and analysis group (putative malignant or normal epithelium), with normal-tissue epithelial cells used as controls and GEO dataset modeled as the batch. Conditions containing fewer than 20 cells were excluded, and up to 400 cells were retained per condition. The final model included 25,810 cells, 106 conditions (42 malignant and 64 normal), and 158 genes. Model fitting, cross-refit stability, permutation, and condition-level pseudobulk comparisons are described in **Supplementary Methods SM7**.

### 2.14 Immune-context, spatial, and ICI-treated cohort analyses

In the five-GEO atlas, patients with at least 20 malignant epithelial cells and 20 cells in the relevant immune compartment were included. Mean MP7 usage and pseudobulk SERINC2 logCPM were calculated in malignant epithelial cells. For 14 prespecified ligand-receptor pairs, malignant-epithelial ligand and tumor-immune receptor expression were summarized as pseudobulk logCPM and combined using the geometric mean. Associations were tested after rank transformation and adjustment for dataset using 10,000 within-dataset permutations, followed by Benjamini-Hochberg correction.

Spatial analysis used four Visium CytAssist FFPE sections from two patients with paired primary CRC and liver metastasis in GSE267401 (37). Counts were normalized to 10,000 per spot and log-transformed. MP7 scores were calculated from the standardized expression of the top 100 MP7 genes after excluding SERINC2. Epithelial, immune, cytotoxicity, T-cell-inflamed, and myeloid-inflammation scores were calculated similarly. Each section was partitioned into 30 coordinate-based blocks using k-means clustering. Partial rank correlations were adjusted for epithelial score and library size; the SERINC2-MP7 analysis was additionally adjusted for immune score. Significance was evaluated using 10,000 block permutations with Benjamini-Hochberg correction across section-wise tests.

The ICI analysis used pretreatment single-cell counts, author-provided annotations, and clinical response data from GSE236581 (38). Twenty patients with CRC were included (11 complete responses, six partial responses, and three stable diseases); complete and partial responses were classified as responders. Immune-state fractions were calculated among tumor immune cells, and associations with the ordered SD-PR-CR response were assessed by Spearman correlation. Nineteen patients with sufficient tumor epithelial cells were included in pseudobulk analysis. MP7 activity was calculated from the mean z-scored logCPM of its top 100 genes after excluding SERINC2, and SERINC2 expression was analyzed separately. Binary response comparisons used exact patient-label permutations, AUCs, rank-biserial effects, and 10,000 bootstrap replicates. P values were adjusted within each analysis family using the Benjamini-Hochberg method.

### 2.15 Statistical analysis and visualization

All tests were two-sided unless specified otherwise. Continuous-group comparisons used Wilcoxon rank-sum tests when appropriate, and associations among continuous scores used Spearman correlation. Survival analyses used Kaplan-Meier estimation, log-rank testing, and Cox proportional-hazards models. Multiple testing used the Benjamini-Hochberg procedure or the FDR output of the corresponding enrichment tool.

## 3 Results

### 3.1 Integrated single-cell atlas reveals tissue-state-associated remodeling of the CRC ecosystem

**Figure 2:**
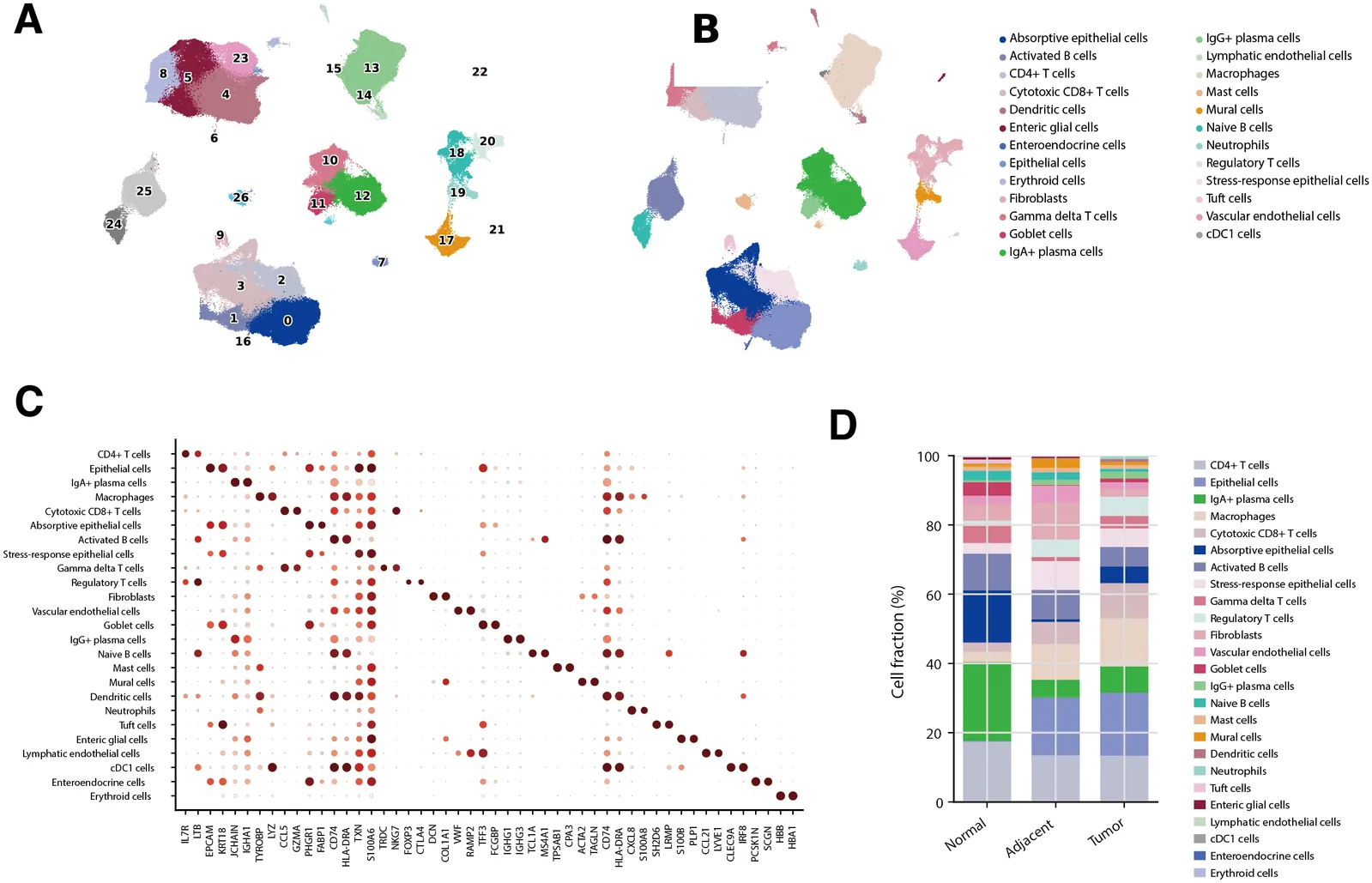
Integrated single-cell atlas of colorectal cancer and related tissues. **(A)** UMAP of 425,402 scVI-integrated cells colored by 27 Leiden clusters. **(B)** UMAP colored by 25 annotated cell types. **(C)** Dot plot of representative canonical markers; dot size indicates the fraction of expressing cells and color indicates scaled mean expression. **(D)** Cell-type distributions in normal, adjacent, and tumor tissues. Fractions are descriptive summaries of the observed cells in each tissue category.

To better understand the TME landscape of CRC, we integrated five scRNA-seq datasets. After quality control, 425,402 cells from 119 patients and 199 samples were retained. scVI integration followed by Leiden clustering identified 27 clusters in the integrated latent space (**Figure 2A**). The major cell-type structure remained consistent under alternative batch specifications (**Supplementary Figure 1**).

Based on canonical and cluster-specific markers, these clusters were assigned to 25 cell types spanning epithelial, immune, and stromal compartments (**Figures 2B, C**). The epithelial compartment included absorptive and stress-response epithelial cells, goblet cells, enteroendocrine cells, and tuft cells. Major immune populations included CD4+ and cytotoxic CD8+ T cells, B cells, plasma cells, macrophages, dendritic cells, and neutrophils, whereas the stromal compartment included fibroblasts, vascular and lymphatic endothelial cells, mural cells, and enteric glial cells. Their lineage-specific marker expression supported the annotations (**Figure 2C**). Cell distributions differed among tumor, adjacent, and normal tissues (**Figure 2D**). Tumors contained relatively higher proportions of generic epithelial cells, macrophages, and cytotoxic CD8+ T cells, whereas normal tissues contained more absorptive epithelial cells and IgA+ plasma cells. Fibroblasts and macrophages were more prominent in adjacent tissues. In summary, the integrated atlas delineated the major cellular compartments and their tissue-associated changes in CRC.

### 3.2 CNV inference identifies putative malignant epithelial cells with MYC/mTORC1 and biosynthetic activation

**Figure 3:**
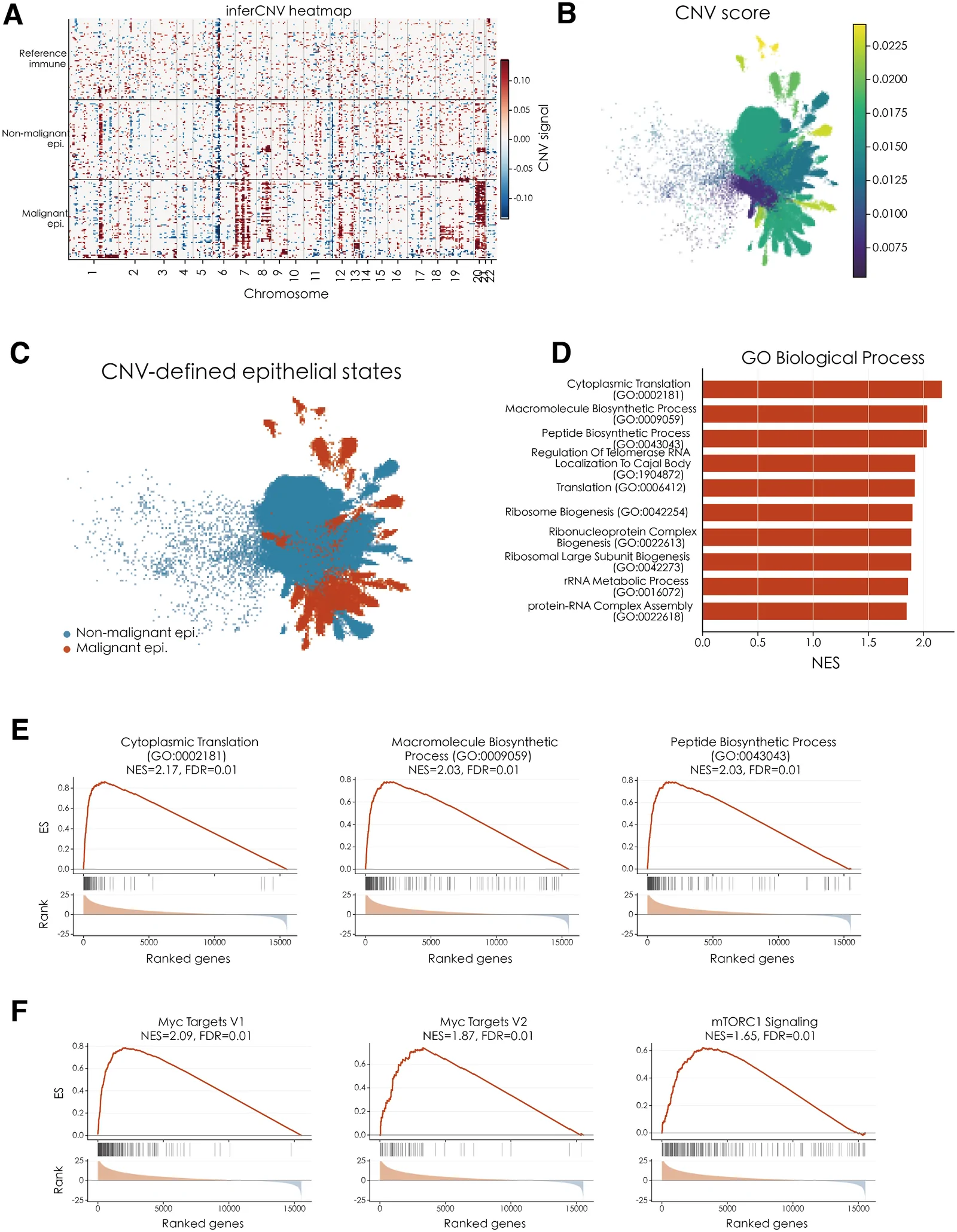
CNV-based identification and functional characterization of putative malignant epithelial cells. **(A)** Genome-ordered inferCNV heat map using immune cells as the reference. **(B)** UMAP colored by epithelial-cell CNV score. **(C)** UMAP showing the CNV score-based assignments of putative malignant and nonmalignant epithelial cells. **(D)** Representative Gene Ontology Biological Process enrichment results for genes upregulated in putative malignant epithelial cells. **(E)** Preranked GSEA curves for cytoplasmic translation, macromolecule biosynthetic process, and peptide biosynthetic process. **(F)** Hallmark GSEA curves for MYC Targets V1, MYC Targets V2, and mTORC1 Signaling. A positive NES indicates enrichment toward the malignant-cell end of the ranked list. NES, normalized enrichment score; FDR, false discovery rate.

We next examined the epithelial compartment by inferring broad copy-number-associated expression changes, using immune cells as the reference. The inferCNV heat map showed coordinated expression shifts across contiguous chromosomal regions in a subset of epithelial cells, whereas the immune reference remained comparatively stable (**Figure 3A**). CNV scoring identified 22,848 putative malignant epithelial cells, including 22,770 from tumors, 75 from adjacent tissues, and 3 from normal tissues (**Figures 3B, C**). Because this classification was based on RNA expression, these cells are referred to as putative malignant epithelial cells throughout the study.

Genes upregulated in the putative malignant cells were enriched in translation, macromolecule biosynthesis, peptide biosynthesis, and ribosome biogenesis (**Figure 3D**). Preranked GSEA showed positive enrichment of cytoplasmic translation (NES = 2.17, FDR = 0.01), macromolecule biosynthetic process (NES = 2.03, FDR = 0.01), and peptide biosynthetic process (NES = 2.03, FDR = 0.01) (**Figure 3E**). Hallmark analysis also identified enrichment of MYC Targets V1 (NES = 2.09, FDR = 0.01), MYC Targets V2 (NES = 1.87, FDR = 0.01), and mTORC1 Signaling (NES = 1.65, FDR = 0.01) (**Figure 3F**).

Patient-aware CNV calibration and scMalignantFinder were used to assess the malignant-cell assignments (**Supplementary Figure 2**). scMalignantFinder supported 22,555 tumor-derived cells (98.7% of the primary population), and the five-state composition was nearly unchanged after filtering (total variation distance=0.0047). The SERINC2-MP7 correlation was also similar in the primary and classifier-supported populations (Spearman ρ=0.336 and 0.340, respectively). The two stricter definitions retained 92.2% and 94.2% of MP7-dominant cells, and both the five-state composition and the SERINC2–MP7 correlation changed little after filtering.

### 3.3 Five malignant epithelial states display distinct transcriptional identities and cell-cycle-sensitive CytoTRACE2 scores

**Figure 4:**
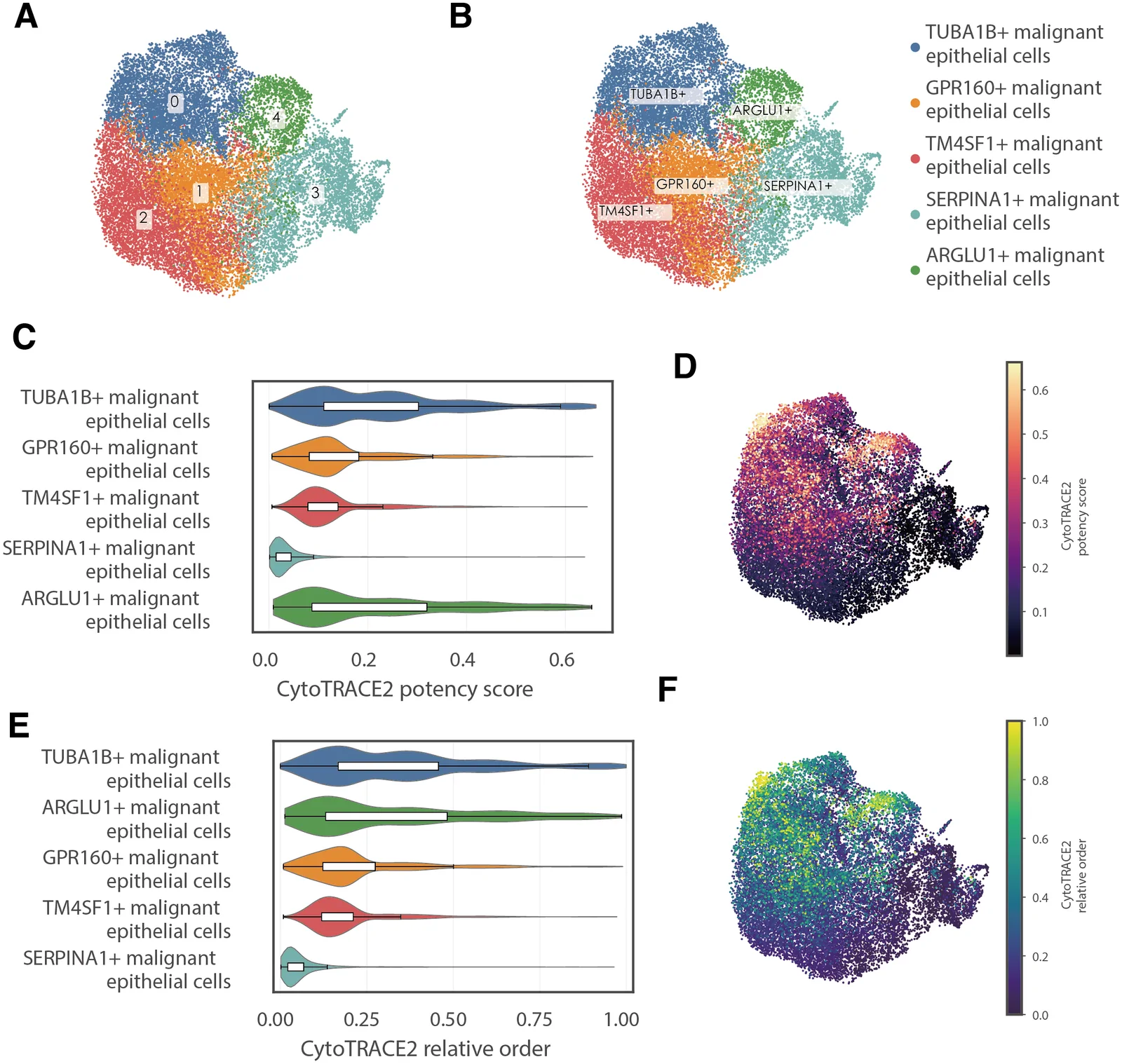
CytoTRACE2-derived scores across malignant epithelial states. **(A)** UMAP of malignant epithelial cells colored by Leiden cluster. **(B)** UMAP colored by the five annotated malignant epithelial states. **(C)** CytoTRACE2 model-defined potency-score distributions shown as violin plots with embedded box plots. **(D)** UMAP projection of the CytoTRACE2 score. **(E)** CytoTRACE2 relative-order distributions shown as violin plots with embedded box plots. **(F)** UMAP projection of relative order. These scores are descriptive outputs and do not imply a shared cross-patient developmental trajectory or clonal direction.

Reclustering of the putative malignant epithelial cells identified five states, designated TUBA1B+, GPR160+, TM4SF1+, SERPINA1+, and ARGLU1+ malignant epithelial cells (**Figures 4A, B**). The TUBA1B+ state expressed proliferation-associated genes, including UBE2C, BIRC5, PTTG1, and MKI67. The TM4SF1+ state was marked by TM4SF1, AREG, CXCL2, CXCL3, and PLAUR, whereas the SERPINA1+ state expressed secretory epithelial genes such as TFF1, TFF3, REG4, and MUC1. The ARGLU1+ state expressed JUN, SOX4, VEGFA, and HSPA5, and the GPR160+ cells formed a separate intestinal epithelial state.

All five states were detected in each of the five GEO datasets, although their relative abundance varied among patients and cohorts (**Supplementary Figures 3A, B**). Under the prespecified detection criteria of at least 10 cells and at least 1% of a patient’s malignant epithelial cells, individual states were represented in 16–34 patients and four or five datasets. Representation was lowest in GSE144735, which contained only 121 malignant epithelial cells. Each state-marker score remained higher in its corresponding state in models accounting for dataset and patient effects (effect estimates = 0.212-1.180; all FDR< 0.001; **Supplementary Figure 3C**). Leave-one-dataset-out classification achieved an overall patient-state-pseudobulk balanced accuracy of 0.677 and a macro-F1 of 0.673, compared with a five-class chance level of 0.20 (**Supplementary Figure 3D**). These findings support the recurrence of the five transcriptional states while showing variation in their prevalence across patients and datasets.

CytoTRACE2 scores differed among the five states, with the highest median score in TUBA1B+ cells (0.204) and the lowest in SERPINA1+ cells (0.026) (**Figures 4C–F**). After removing 94 detected S- and G2/M-phase genes, the original and cell-cycle-depleted scores remained correlated across cells (Spearman’s ρ = 0.928; **Supplementary Figures 3E, F**), but the median difference between TUBA1B+ cells and the other states decreased from 0.099 to 0.040 in 33 matched patients (**Supplementary Figure 3G**). The TUBA1B+ effect was no longer significant when S- and G2/M-phase scores were included in the model for either the original score (β = 0.0167, P = 0.467) or the cell-cycle-depleted score (β = 0.0065, P = 0.732; **Supplementary Figure 3H**). All five transcriptional states recurred across patients and datasets, although their prevalence varied.

### 3.4 pySCENIC reveals state-specific regulon activity across malignant epithelial states

**Figure 5:**
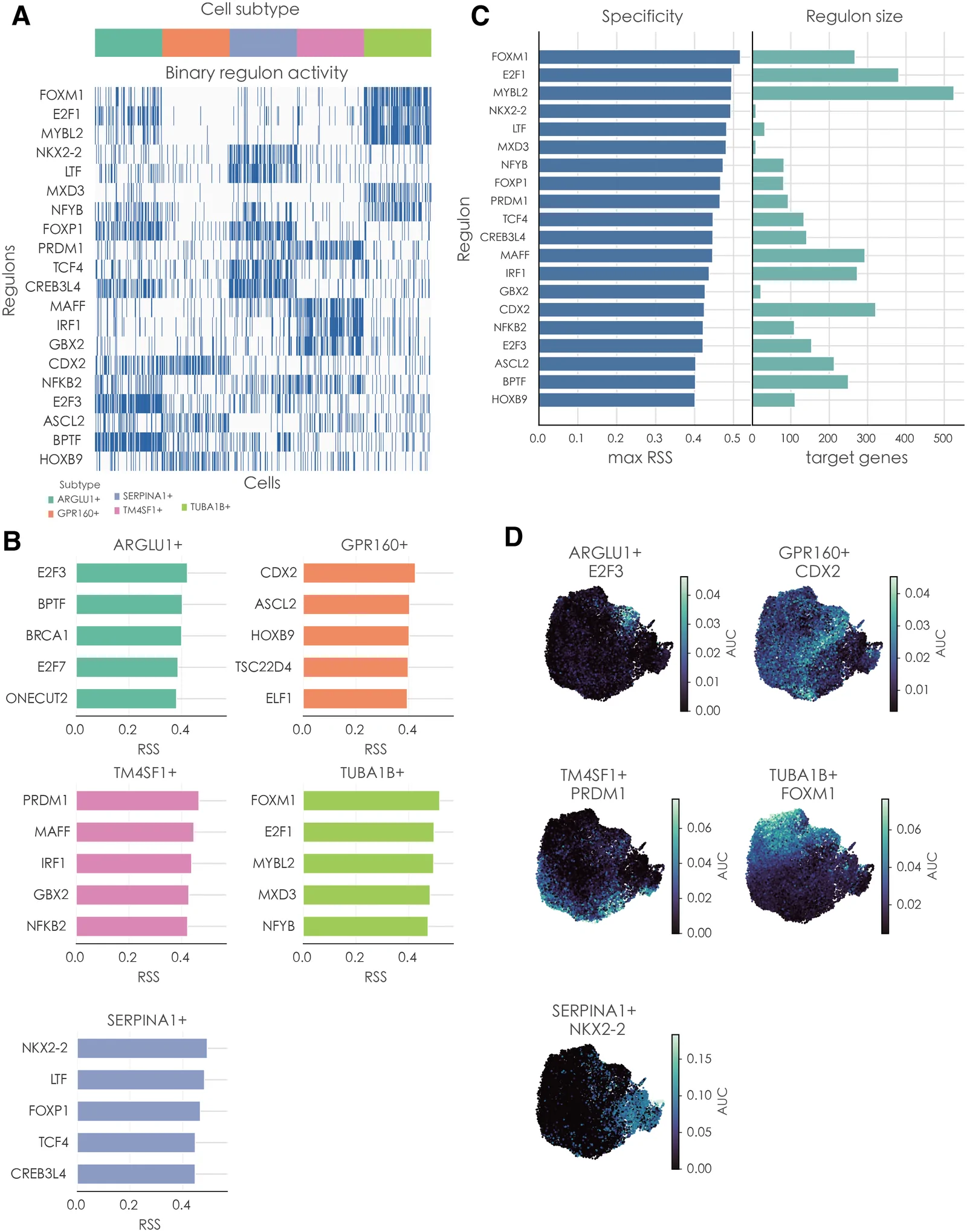
State-associated transcriptional regulation inferred by pySCENIC. **(A)** Binarized regulon-activity heat map with cells ordered by malignant epithelial state. **(B)** Top five regulons ranked by regulon specificity score (RSS) for each state. **(C)** Maximum RSS and regulon target-gene number for representative state-associated regulons. **(D)** AUCell activity projected onto the malignant-epithelial UMAP for E2F3, CDX2, PRDM1, FOXM1, and NKX2–2 regulons. Cross-dataset reconstruction and within-patient recurrence are shown in **Supplementary Figure 4**.

pySCENIC analysis identified distinct regulon-activity profiles across the five malignant epithelial states (**Figure 5A**). RSS analysis associated the proliferative TUBA1B+ state with FOXM1, E2F1, MYBL2, MXD3, and NFYB regulons; FOXM1 showed the highest RSS (0.517), and MYBL2 contained the largest target-gene set among the displayed regulons (**Figures 5B, C**). The GPR160+ state was associated with CDX2, ASCL2, and HOXB9; the TM4SF1+ state with PRDM1, MAFF, IRF1, and NFKB2; the SERPINA1+ state with NKX2-2, LTF, and FOXP1; and the ARGLU1+ state with E2F3, BPTF, and E2F7 (**Figures 5B, D**).

Network reconstruction in four individual datasets and within-patient AUCell analysis were then used to assess the recurrence of the 25 state-associated regulons (**Supplementary Figure 4**). Eighteen regulons met the network-overlap criterion, 16 showed recurrent state-associated activity across patients, and 15 met both criteria. These included E2F3, BPTF, and E2F7 for ARGLU1+ cells; CDX2, HOXB9, and ELF1 for GPR160+ cells; FOXP1 for SERPINA1+ cells; PRDM1, MAFF, IRF1, and NFKB2 for TM4SF1+ cells; and FOXM1, E2F1, MYBL2, and NFYB for TUBA1B+ cells. All 15 associations remained significant in models that included within-patient CNV scores (FDR< 0.05), with a median absolute change of 0.00073 in the state coefficient. Thus, recurrent regulon activity was detected for all five states, with the largest reproduced regulon sets observed in the TUBA1B+ and TM4SF1+ states.

### 3.5 cNMF identifies eight malignant epithelial metaprograms, including a secretory MP7 program recurrent in a subset of tumors

**Figure 6:**
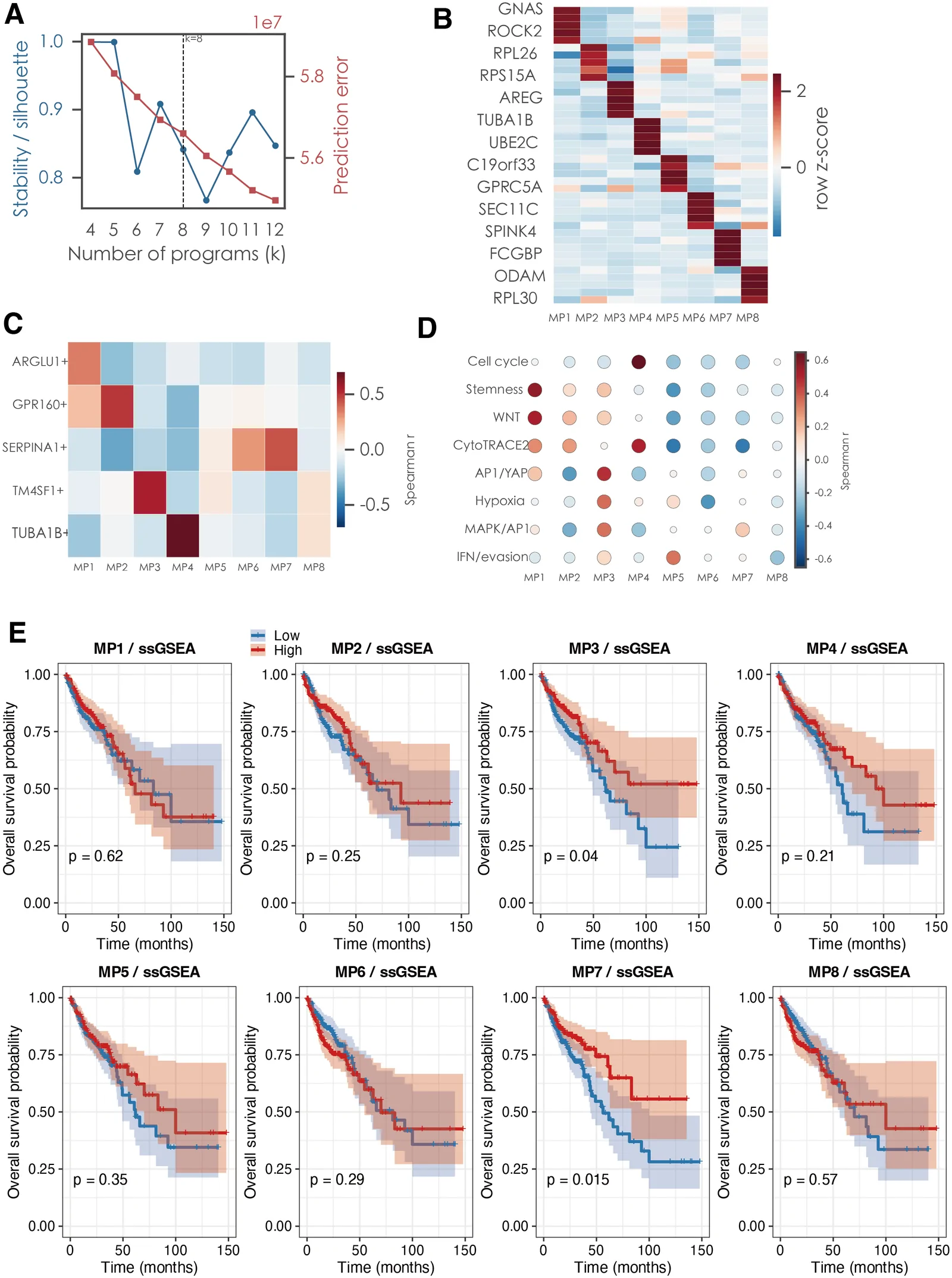
cNMF metaprograms and their clinical associations. **(A)** Stability and reconstruction error across candidate factorization ranks. **(B)** Representative top-weighted genes for MP1-MP8. **(C)** Correlation of metaprogram usage with malignant epithelial states. **(D)** Correlation between metaprogram usage and predefined functional scores; color indicates direction and magnitude and point size indicates statistical strength. **(E)** Kaplan-Meier overall-survival curves for MP1-MP8 in TCGA-COAD, stratified at the median ssGSEA score. Patient-level metaprogram recurrence is shown in **Supplementary Figure 5**; epithelial-lineage comparisons and single-cell-to-bulk transfer analyses are shown in **Supplementary Figures 6A–C**, **6D–F**, respectively.

cNMF analysis of the putative malignant epithelial cells supported a rank of K = 8, yielding eight metaprograms designated MP1-MP8 (**Figures 6A, B**). Several metaprograms were associated with specific epithelial states and functional signatures. MP1 was associated with ARGLU1+ cells (Spearman’s ρ = 0.353) and correlated with the stemness/WNT cancer-stem-cell score (ρ = 0.565). MP2 was most strongly associated with GPR160+ cells (ρ = 0.501). MP3 was enriched in TM4SF1+ cells (ρ = 0.555) and correlated with the oncofetal AP1/YAP plasticity score (ρ = 0.481). MP4 was associated with TUBA1B+ cells (ρ = 0.708) and showed the strongest correlation with the cell-cycle/proliferation score (ρ = 0.760) (**Figures 6C, D**).

MP7 was preferentially expressed in SERPINA1+ malignant epithelial cells (ρ = 0.440; odds ratio = 12.34). Its highest-weight genes included SPINK4, MUC1, NNMT, FCGBP, MUC5B, REG4, REG1B, REG1A, TFF3, and AGR2, defining a mucinous secretory program. MP7 usage was negatively correlated with canonical WNT signaling (ρ = −0.201), cell-cycle/proliferation (ρ = −0.204), and CytoTRACE2 score (ρ = −0.439), distinguishing it from the proliferation-associated MP4.

Patient-level cNMF analysis showed different degrees of recurrence among the metaprograms (**Supplementary Figure 5**). MP4- and MP5-like programs were recovered in 26 of 26 and 24 of 26 evaluable patients, respectively. An MP7-like program was identified in 9 of 26 patients across three datasets; six patients reproduced MP7 at two or more factorization ranks, and five retained a reciprocal-best match. Among the best MP7-like matches, the median gene-weight correlation was 0.263 and the median overlap among the top 100 genes was 24 genes. TFF3 and LGALS4 recurred in nine and seven of the nine matched patients, respectively. These findings indicate that MP7 recurs in a subset of CRC tumors rather than representing a uniformly expressed malignant program.

Comparison with nonmalignant epithelial populations showed that MP7 retained a strong goblet-lineage component (**Supplementary Figures 6A–C**). Median MP7 scores were 0.880 in normal goblet cells, 1.053 in tumor-associated nonmalignant goblet cells, and 1.174 in malignant MP7-dominant cells, compared with 0.249 in malignant non-MP7 cells; all three comparisons with malignant non-MP7 cells remained significant in models accounting for dataset and patient effects (all FDR< 3 × 10^-26^). The MP7 top-100 gene profile was also highly similar between normal goblet cells and malignant MP7-dominant cells (ρ = 0.834, P = 4.36 × 10^-27^). MP7 therefore represents a secretory/goblet-lineage program expressed by a subset of malignant epithelial cells rather than a tumor-specific transcriptional program.

The single-cell-derived MP7 score correlated with mean MP7 usage in malignant-cell patient pseudobulks (ρ = 0.353, P = 0.000924; **Supplementary Figures 6D–F**). When projected into 389 TCGA-COAD tumors, MP7 showed the strongest nominal survival association among the eight metaprograms: patients with high MP7 scores had longer overall survival in the median-split analysis (HR = 0.599, 95% CI = 0.394-0.910; log-rank P = 0.015; **Figure 6E**). However, the continuous MP7 score was not significantly associated with survival either before adjustment (HR per SD = 0.849, P = 0.095) or after adjustment for clinical variables and tumor purity (HR = 0.858, P = 0.304). The bulk MP7 score correlated strongly with normal goblet-lineage expression (ρ = 0.794) and inversely with tumor purity (ρ = −0.221, P = 9.96 × 10^-6^), indicating that its TCGA projection was influenced by epithelial composition and did not establish MP7 as an independent prognostic factor. MP7 therefore recurred in a subset of CRC tumors and was not uniformly represented across the cohort.

### 3.6 An exploratory MP7-derived 17-gene score shows limited prognostic discrimination

**Figure 7:**
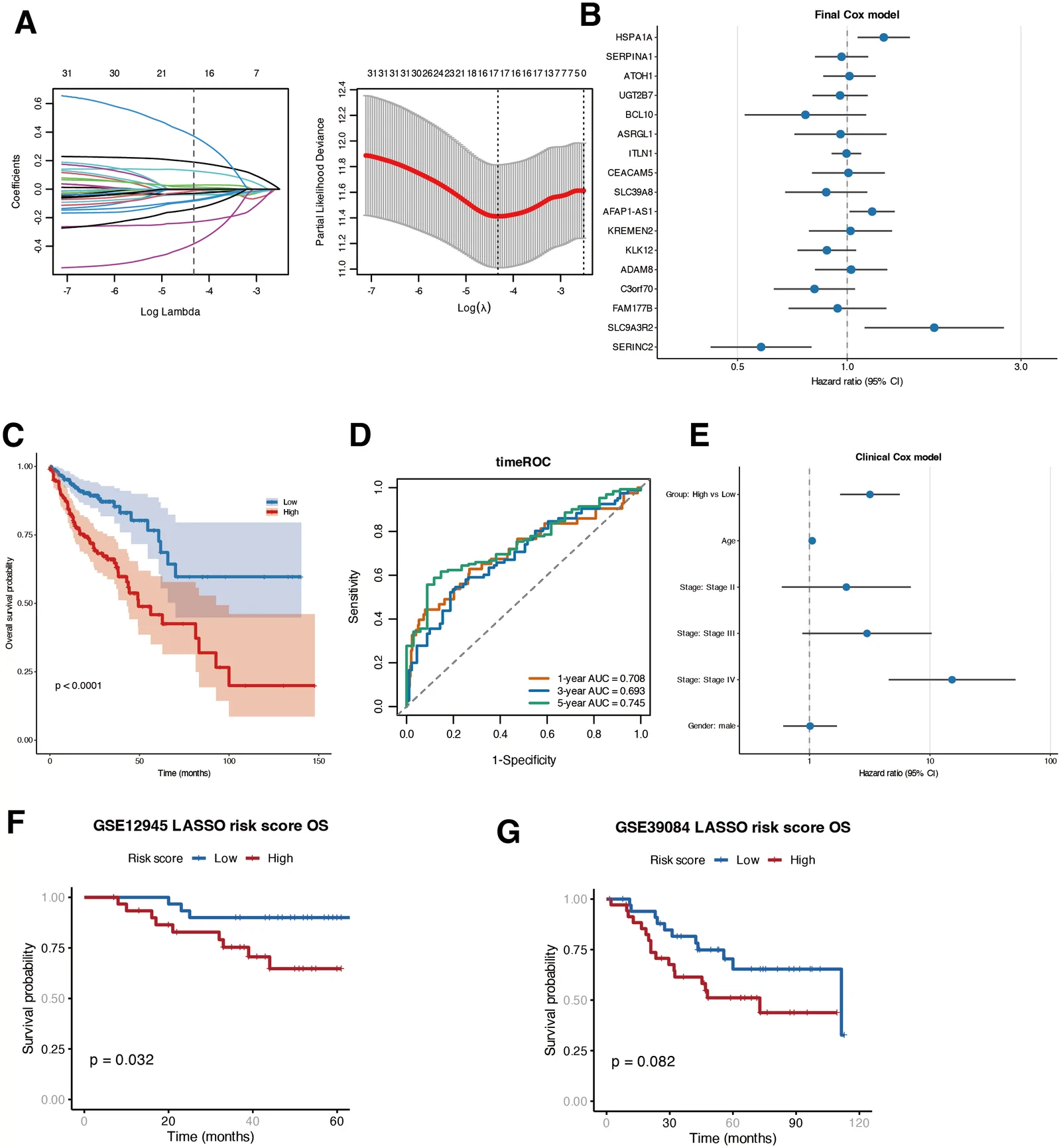
Construction and performance assessment of the exploratory MP7-derived 17-gene survival score. **(A)** LASSO-Cox coefficient paths and cross-validation curve. **(B)** Forest plot of the TCGA-derived 17-gene Cox model. **(C)** Kaplan-Meier overall-survival curves in TCGA-COAD. **(D)** Time-dependent ROC curves at 1, 3, and 5 years. **(E)** Multivariable Cox analysis adjusted for age, sex, and pathological stage. **(F)** Assessment in GSE12945. **(G)** Assessment in GSE39084. Nested cross-validation and assessments in the larger external cohorts are shown in **Supplementary Figures 7A–D**.

We next explored whether the 300 highest-ranking MP7 genes could be combined into a survival score in TCGA-COAD. Of the 298 genes detected, 32 were associated with overall survival in univariable Cox analysis, and LASSO-Cox regression retained 17 genes: HSPA1A, SERPINA1, ATOH1, UGT2B7, BCL10, ASRGL1, ITLN1, CEACAM5, SLC39A8, AFAP1-AS1, KREMEN2, KLK12, ADAM8, C3orf70, FAM177B, SLC9A3R2, and SERINC2 (**Figures 7A, B**). In the TCGA derivation cohort, the high-risk group had shorter overall survival than the low-risk group (HR = 2.80, 95% CI = 1.80-4.36, P = 5.0×10^-6^; **Figure 7C**). The association remained significant after adjustment for age, sex, and simplified pathological stage (HR = 3.18, 95% CI = 1.80-5.62, P = 6.96×10^-5^; **Figure 7E**). The corresponding time-dependent AUCs at 1, 3, and 5 years were 0.708, 0.693, and 0.745, respectively (**Figure 7D**).

The performance observed in the derivation cohort was not maintained during nested cross-validation. When gene screening and model fitting were repeated within 20 × five-fold nested cross-validation, the median held-out C-index was 0.533 (IQR = 0.515-0.553), and the median held-out HR per standard-deviation increase in the score was 1.080 (**Supplementary Figure 7A**). In repeated held-out comparisons, the median C-index was 0.664 for the 17-gene score, 0.772 for the clinical model, and 0.766 for the combined model. Calibration and Brier-score analyses also showed no consistent improvement after adding the gene score to the clinical variables (**Supplementary Figure 7B**).

In the two smaller microarray cohorts, the score separated survival groups in GSE12945 (log-rank P = 0.032), whereas the association did not reach significance in GSE39084 (P = 0.082; **Figures 7F, G**). In the larger GSE39582 and GSE17536 cohorts, 15 of the 17 genes were available. The adjusted HR per standard-deviation increase in the score was 1.217 in GSE39582 (95% CI = 1.068-1.386, P = 0.0032) and 1.280 in GSE17536 (95% CI = 1.003-1.634, P = 0.0475) (**Supplementary Figure 7C**). However, the score-only C-indices were 0.570 and 0.543, and adding the score produced little or no improvement over the corresponding clinical models (**Supplementary Figure 7D**). Thus, although the direction of the survival association was reproduced in the larger cohorts, the 17-gene score showed limited discrimination and did not improve prediction beyond standard clinical variables.

### 3.7 Survival-learning analyses prioritize HSPA1A, AFAP1-AS1, and SERINC2

**Figure 8:**
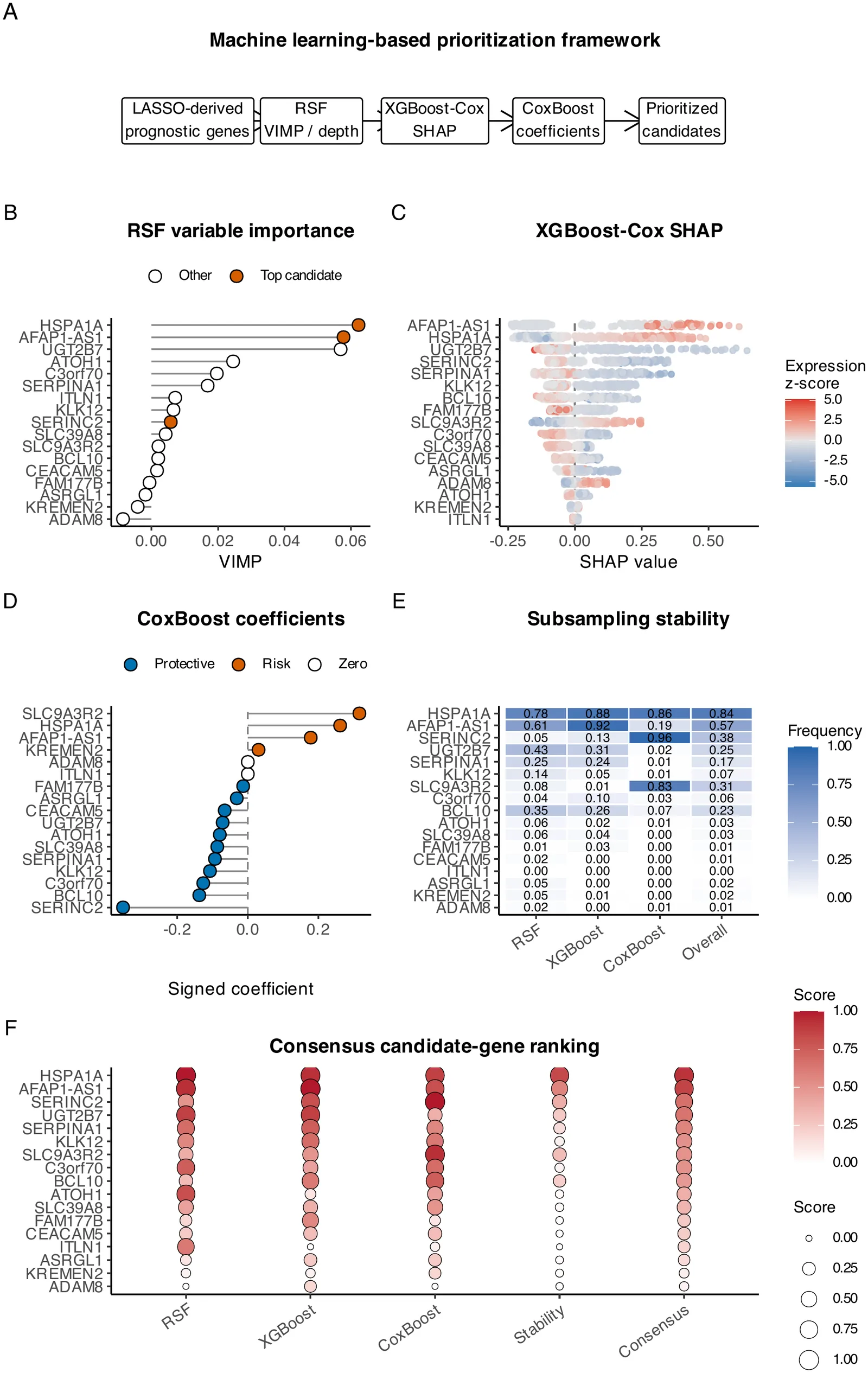
Multi-algorithm within-cohort ranking of MP7-derived candidate genes. **(A)** Candidate-ranking workflow. **(B)** Random-survival-forest variable importance. **(C)** XGBoost-Cox SHAP summary plot. **(D)** Signed CoxBoost coefficients. **(E)** Subsampling selection frequencies. **(F)** Consensus candidate-gene ranking integrating random survival forest, XGBoost-Cox/SHAP, CoxBoost, and stability scores. All rankings were derived from the same TCGA-COAD cohort and were used for candidate prioritization.

The 17 genes retained by LASSO-Cox were further ranked using random survival forests, XGBoost-Cox, CoxBoost, and repeated subsampling in TCGA-COAD (**Figures 8A–F**). The consensus analysis placed HSPA1A, AFAP1-AS1, and SERINC2 in the first, second, and third positions, respectively. Support for SERINC2 varied among algorithms: it ranked ninth by random-survival-forest importance, fourth by XGBoost-Cox SHAP values, and first by the absolute CoxBoost coefficient. The consensus ranking was therefore used to prioritize genes for further investigation within the MP7-derived candidate set.

In the TCGA derivation model, higher SERINC2 expression was associated with better overall survival (HR = 0.580, P = 0.000823). This association was not reproduced in GSE39582 (adjusted HR per SD = 1.008, 95% CI = 0.867-1.171, P = 0.921) or GSE17536 (adjusted HR per SD = 0.931, 95% CI = 0.738-1.176, P = 0.549). The fixed-effect meta-analysis was also nonsignificant (HR = 0.985, 95% CI = 0.868-1.117, P = 0.810; I^2^ = 0%; **Supplementary Figures 7E, F**). Thus, the survival analyses did not support SERINC2 as an independent prognostic marker, and it was taken forward as an MP7-derived candidate for functional characterization.

### 3.8 SERINC2-targeting shRNAs increase wound closure and Transwell invasion in CRC cells

**Figure 9:**
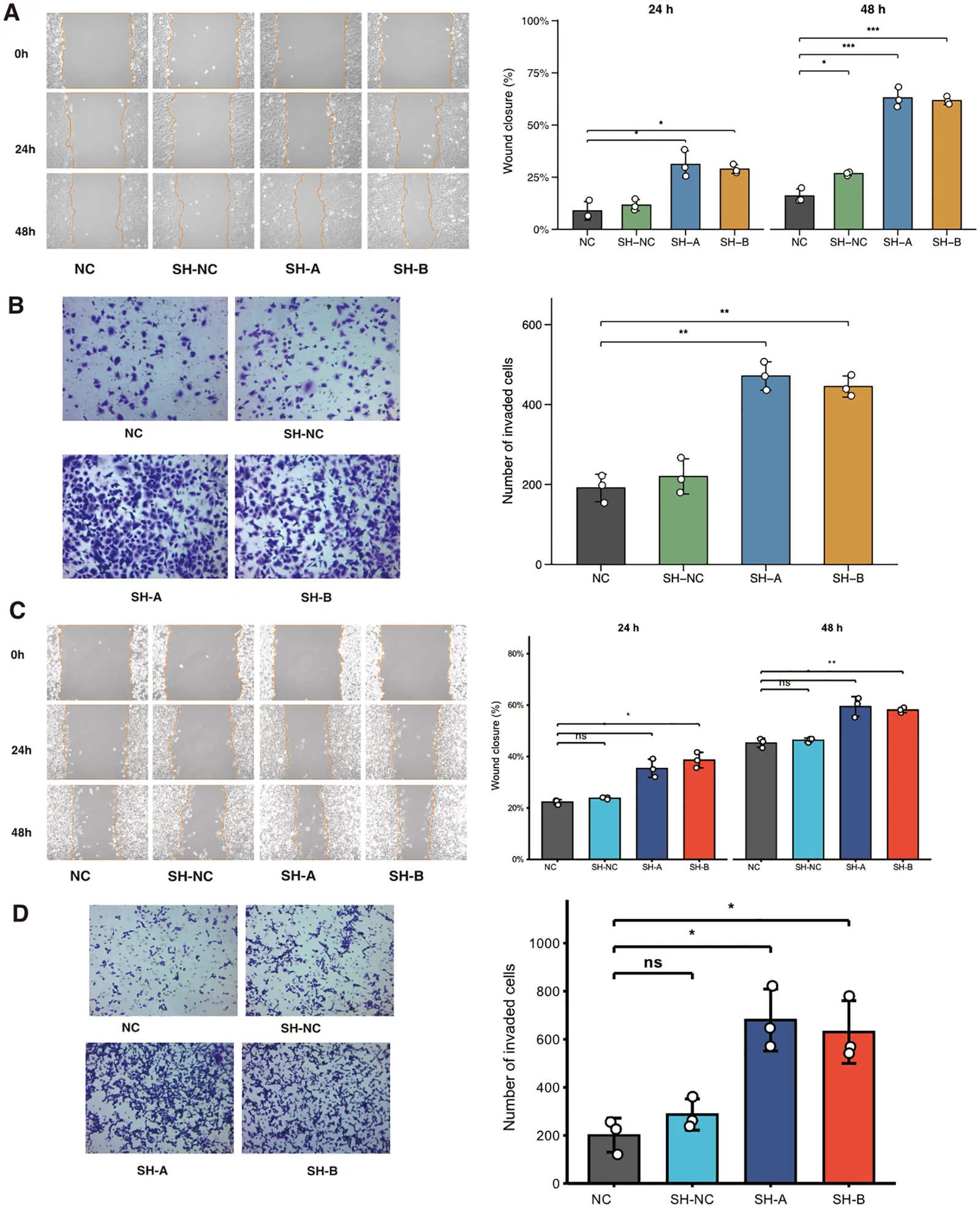
Effects of SERINC2-targeting shRNAs on wound closure and Transwell invasion in SW480 and RKO colorectal cancer cells. **(A)** Representative wound-healing images at 0, 24, and 48 h and quantification of wound closure in SW480 cells. **(B)** Representative Matrigel-coated Transwell images and invaded-cell counts at 24 h in SW480 cells. **(C)** Representative wound-healing images at 0, 24, and 48 h and quantification of wound closure in RKO cells. **(D)** Representative Matrigel-coated Transwell images and invaded-cell counts at 24 h in RKO cells. NC denotes the untreated control, SH-NC denotes the non-targeting shRNA control, and SH-A and SH-B denote the two SERINC2-targeting shRNAs. RNA-level SERINC2 knockdown was assessed only in SW480 cells (**Supplementary Figure 8**). Points show individual measurements; bars and error bars indicate the mean and SD. Comparisons were made with NC using replicate-blocked linear models with Dunnett adjustment. ns, not significant; *P<0.05; **P<0.01; ***P<0.001.

RT-qPCR confirmed efficient SERINC2 knockdown in SW480 cells. Across three independent experiments, SH-A and SH-B each reduced SERINC2 mRNA expression by approximately 94% relative to SH-NC (**Supplementary Figure 8**). In SW480 cells, both shRNAs increased wound closure at 24 and 48 h and increased the number of cells invading through Matrigel-coated Transwell membranes at 24 h (**Figures 9A, B**). RKO cells transduced with the same SERINC2-targeting constructs showed similar phenotypes, although target reduction was not measured in this cell line. At 24 h, mean wound closure was 23.8% in SH-NC cells and 35.4% and 38.6% in SH-A and SH-B cells, respectively; wound closure remained higher at 48 h (**Figure 9C**). Both constructs also increased Transwell invasion at 24 h (**Figure 9D**).

### 3.9 D-SPIN identifies stable local SERINC2 couplings

D-SPIN was used to characterize the epithelial expression context of SERINC2. Across 15 refits, whole-matrix Spearman correlations with the main fit ranged from 0.820 to 0.884, and the mean absolute coupling of the 20 strongest SERINC2 edges was 4.73-fold higher than the within-condition permutation median (empirical P = 0.032; **Supplementary Figure 9**). Seventeen edges met the cross-refit stability criterion; stable positive partners included CEACAM7, LYPD8, TFF1, GUCA2A, FABP1, and SERPINA1, whereas UBE2C showed a negative coupling. Sixteen of the 17 stable edges had the same direction in the adjusted condition-level pseudobulk analysis, although SERINC2 ranked at only the 3.3rd percentile for coupling strength among expression-matched genes. SERINC2 showed reproducible local couplings but low global coupling strength; this analysis did not test causality.

### 3.10 Immune and spatial associations of MP7 and SERINC2 in CRC

**Figure 10:**
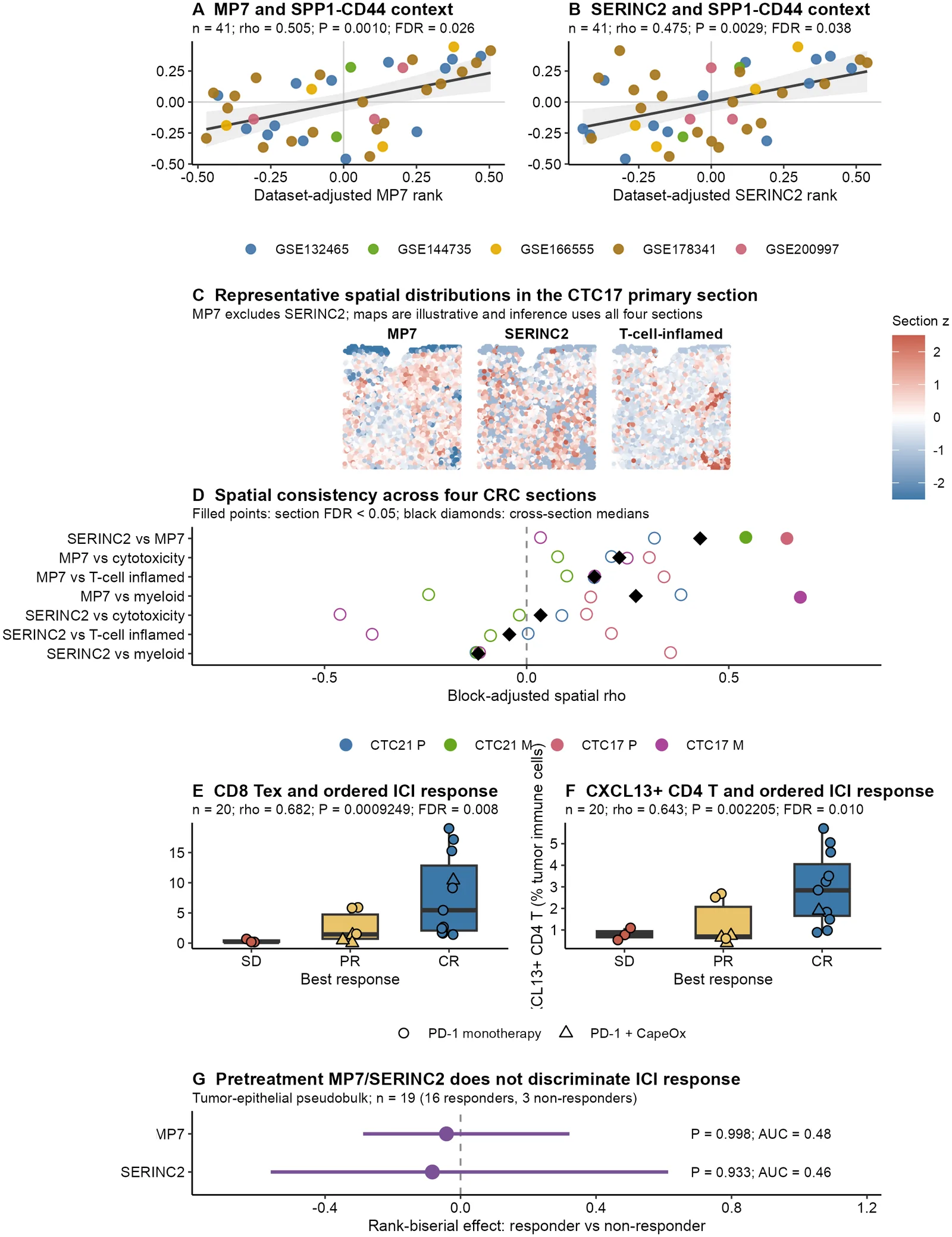
Immune context, spatial localization, and ICI response in relation to MP7 and SERINC2. **(A, B)** Patient-level associations of malignant-epithelial MP7 activity or SERINC2 pseudobulk expression with the SPP1-CD44 co-expression score in the five-GEO atlas. Points indicate patients and colors indicate datasets; axes show dataset-adjusted rank residuals, and P values were obtained by within-dataset permutation with Benjamini-Hochberg correction. **(C)** Representative GSE267401 spatial maps of the MP7 score excluding SERINC2, SERINC2 expression, and the T-cell-inflamed program in the CTC17 primary CRC section. **(D)** Block-adjusted spatial rank correlations across four paired primary or metastatic sections. Filled points denote section-level FDR<0.05; diamonds summarize the median across sections. **(E, F)** Pretreatment CD8 Tex and CXCL13-positive CD4 T-cell fractions across ordered SD, PR, and CR categories in 20 ICI-treated patients with CRC. Shapes distinguish anti-PD-1 monotherapy from PD-1 plus CapeOx. **(G)** Rank-biserial response effects and bootstrap 95% confidence intervals for MP7 and SERINC2 in 19 evaluable epithelial samples; exact permutation P values and AUCs are shown.

We first examined whether epithelial MP7 activity and SERINC2 expression were associated with immune features in the integrated five-dataset atlas. Among 41 patients with sufficient malignant epithelial and immune cells, patient-level MP7 activity correlated positively with the SPP1-CD44 co-expression score after adjustment for dataset (ρ = 0.505, permutation P = 0.0010, FDR = 0.026; **Figure 10A**). SERINC2 expression showed a similar association (ρ = 0.475, permutation P = 0.0029, FDR = 0.038; **Figure 10B**).

Spatial analysis of four sections from GSE267401 showed a positive association between SERINC2 expression and the MP7 score calculated without SERINC2 in all four sections (median block-adjusted ρ = 0.429), with two sections reaching FDR< 0.05 (**Figures 10C, D**). MP7 activity was also positively associated with cytotoxicity and T-cell-inflamed programs in all four sections, with median correlations of 0.229 and 0.168, respectively, although none of the individual section-level tests remained significant after FDR correction. The association between MP7 and the myeloid-inflammation program was positive in three sections (median ρ = 0.270), with one section reaching FDR< 0.05. These spatial associations therefore varied in strength among sections.

We next examined pretreatment samples from 20 patients with CRC receiving immune checkpoint inhibitors in GSE236581. The fractions of CD8 Tex and CXCL13+ CD4 T cells increased across the ordered SD, PR, and CR response categories (ρ = 0.682, FDR = 0.0083 and ρ = 0.643, FDR = 0.0099, respectively; **Figures 10E, F**). In the 19 patients with evaluable epithelial cells, neither MP7 activity nor SERINC2 expression discriminated responders from nonresponders (MP7: exact P = 0.998, AUC = 0.48; SERINC2: exact P = 0.933, AUC = 0.46; **Figure 10G**). Thus, the immune and spatial associations of MP7 and SERINC2 did not translate into ICI-response discrimination in this cohort.

## 4 Discussion

By integrating five CRC scRNA-seq datasets, this study identified five malignant epithelial states and eight expression metaprograms with different degrees of recurrence across patients. The five epithelial states were detected across all datasets, although their prevalence varied among patients and cohorts. Among the cNMF programs, MP4 and MP5 were widely recovered, whereas the secretory MP7 program recurred in only a subset of evaluable tumors. MP7 retained a strong goblet-lineage expression pattern and was associated with lower proliferation and WNT-related activity. Further analyses prioritized SERINC2 as an MP7-derived candidate. SERINC2-targeting shRNAs increased wound closure and Transwell invasion in SW480 and RKO cells, with RNA-level knockdown confirmed in SW480 cells.

The recurrence of the five malignant epithelial states across datasets supports the presence of shared transcriptional patterns within the heterogeneous CRC epithelium. Previous single-cell studies have described CRC epithelial states and trajectories, their links to immune organization, and their relationship to molecular classification (6–10). We evaluated state identity at the patient and dataset levels in addition to the integrated clustering analysis. Nevertheless, the states were not equally represented in every tumor, indicating that they should be viewed as recurrent transcriptional phenotypes rather than obligatory components of all CRCs. CytoTRACE2 initially assigned the highest score to the proliferative TUBA1B+ state, but this difference was no longer significant after accounting for S- and G2/M-phase activity, providing no evidence of an independent developmental hierarchy. Similarly, pySCENIC identified state-associated regulons, but only a subset was reproduced across datasets and patients. The recurrent FOXM1/E2F/MYBL2 activity in *TUBA1B*+ cells was consistent with their proliferative phenotype, whereas the regulatory features of the other states were more heterogeneous.

Repeating cNMF within individual tumors showed which programs from the integrated analysis were recovered across patients, extending these earlier state-based descriptions of CRC epithelial heterogeneity. MP7 was distinguished by mucinous and secretory genes, including *SPINK4*, *MUC1*, *FCGBP*, *REG4*, *TFF3*, and *AGR2*, and was recovered in 9 of 26 evaluable patients across three datasets. Its limited recurrence contrasts with the widespread recovery of MP4 and MP5 and suggests that MP7 characterizes a particular subset of tumors. The close similarity between malignant MP7-dominant cells and normal goblet cells further indicates that MP7 is not a tumor-specific program. Instead, it appears to represent a differentiated intestinal secretory program retained or reused by some malignant epithelial cells. This interpretation is consistent with the high MP7 scores observed in both normal and tumor-associated goblet cells. At the same time, most MP7-dominant cells remained within the more stringent malignancy-supported populations, making nonmalignant epithelial contamination an unlikely sole explanation for the program.

Projection of MP7 into bulk tumors illustrated the difficulty of transferring a lineage-related single-cell program to tissue-level expression data. Although high MP7 scores were associated with favorable survival in the median-split TCGA analysis, the continuous association was not significant after adjustment for clinical variables and tumor purity. The strong correlation between bulk MP7 scores and normal goblet-lineage expression further suggests that the bulk signal reflects both malignant-cell state and epithelial composition. The exploratory MP7-derived 17-gene score separated survival groups in the TCGA derivation cohort, but this performance was not maintained during nested cross-validation. In larger external cohorts, the direction of association was partly reproduced, whereas discrimination remained limited and adding the score did not improve prediction beyond clinical variables. RNA-based prognostic biomarkers have also been widely investigated in colorectal and other gastrointestinal cancers. Meta-analyses have reported survival associations for several miRNAs and circular RNAs, but an umbrella review found that most supporting evidence was of low or very low quality (39–41). These findings underscore the need for robust external validation before RNA-based biomarkers are considered for clinical use. The current data do not justify prognostic use of MP7 or the 17-gene score. MP7 nevertheless remains informative as a single-cell secretory program.

Within the MP7-derived 17-gene set, multi-algorithm ranking prioritized SERINC2 for functional analysis. SERINC proteins participate in the incorporation of serine into membrane lipids (42), and previous studies have linked SERINC2 to tumor-cell growth, migration, lipid metabolism, MYC signaling, and immune regulation in lung and cervical cancers (43–45). In the present study, two SERINC2-targeting shRNAs increased wound closure and Transwell invasion in both SW480 and RKO cells, and efficient RNA-level knockdown was confirmed in SW480 cells. The concordant SW480 results are consistent with a possible role for SERINC2 in these phenotypes, whereas the RKO findings require direct confirmation of target reduction. Post-selection D-SPIN analysis linked *SERINC2* to a reproducible local expression context enriched in secretory and differentiated epithelial genes, but *SERINC2* showed low global coupling strength. This supports expression context rather than a direct regulatory mechanism.

The immune analyses connected the epithelial findings to features of the CRC microenvironment. Patient-level MP7 activity and *SERINC2* expression were positively associated with an *SPP1*-*CD44* co-expression score, suggesting that the secretory epithelial program occurs in tumors with a measurable myeloid-epithelial co-expression background. Spatial data further showed consistent *SERINC2*-MP7 associations and weaker, section-dependent relationships between MP7 and cytotoxic, T-cell-inflamed, and myeloid-inflammation programs. These observations support spatial and interpatient covariation but cannot determine whether the epithelial program shapes immune-cell recruitment or instead arises in response to the surrounding microenvironment. In the ICI-treated cohort, response-associated CD8 Tex and *CXCL13*+ CD4 T-cell patterns were detectable, whereas MP7 and *SERINC2* did not distinguish responders from nonresponders. Their association with immune context therefore did not translate into ICI-response prediction.

Several limitations should be considered. The five scRNA-seq datasets differed in tissue sampling, dissociation protocols, sequencing platforms, and clinical annotation, and residual cohort effects may remain despite integration. Malignant-cell assignment was inferred from RNA-based CNV patterns and expression classification rather than matched DNA sequencing or pathological confirmation. CytoTRACE2 scores, pySCENIC regulons, cNMF usage, and D-SPIN couplings are computational estimates and should not be interpreted as direct measurements of developmental direction, transcription-factor binding, or molecular regulation. Patient-level cNMF analysis was restricted to 26 patients with sufficient malignant epithelial cells, and the relationship between MP7 prevalence and tumor location, mucinous histology, MSI, or CMS could not be evaluated consistently. Bulk survival analyses were affected by tissue composition and model overfitting. Although SERINC2-targeting shRNAs produced consistent wound-closure and invasion phenotypes in two CRC cell lines, RNA-level knockdown was confirmed only in SW480 cells. Direct validation of target reduction in RKO cells, protein-level knockdown, rescue experiments, proliferation controls, perturbation-response profiling, and *in vivo* studies will be required to establish specificity and mechanism. Finally, the spatial analysis included four sections from two patients, and the ICI cohort was small, particularly for nonresponders; larger independent cohorts will be required to define the immune relevance of MP7 and *SERINC2*.

In conclusion, this study identified malignant epithelial states and expression programs with varying recurrence across CRC tumors. MP7 was a secretory/goblet-lineage program recovered in a subset of tumors, and SERINC2 emerged as an MP7-derived experimental candidate. SERINC2-targeting shRNAs increased wound closure and Transwell invasion in SW480 and RKO cells, although RNA-level knockdown was confirmed only in SW480 cells. The immune and spatial associations warrant further investigation but did not support MP7 or SERINC2 as predictors of ICI response in the cohort examined.

## Data Availability

Publicly available datasets were analyzed in this study. The original datasets are available from the repositories and under the accession numbers specified in the Materials and Methods section. The analysis code and supporting workflow files generated for this study are deposited in the Zenodo repository, accession number 20827525 (https://doi.org/10.5281/zenodo.20827525).

## References

[1] Bray F Laversanne M Sung H Ferlay J Siegel RL Soerjomataram I . Global cancer statistics 2022: GLOBOCAN estimates of incidence and mortality worldwide for 36 cancers in 185 countries. CA Cancer J Clin. (2024) 74:229–63. doi: 10.3322/caac.21834 38572751

[2] Cancer Genome Atlas Network . Comprehensive molecular characterization of human colon and rectal cancer. Nature. (2012) 487:330–7. doi: 10.1038/nature11252 22810696 PMC3401966

[3] Pagès F Mlecnik B Marliot F Bindea G Ou FS Bifulco C . International validation of the consensus Immunoscore for the classification of colon cancer: a prognostic and accuracy study. Lancet. (2018) 391:2128–39. doi: 10.1016/S0140-6736(18)30789-X 29754777

[4] André T Shiu KK Kim TW Jensen BV Jensen LH Punt CJA . Pembrolizumab in microsatellite-instability-high advanced colorectal cancer. N Engl J Med. (2020) 383:2207–18. doi: 10.1056/NEJMoa2017699 33264544

[5] Guinney J Dienstmann R Wang X de Reyniès A Schlicker A Soneson C . The consensus molecular subtypes of colorectal cancer. Nat Med. (2015) 21:1350–6. doi: 10.1038/nm.3967 26457759 PMC4636487

[6] Joanito I Wirapati P Zhao N Nawaz Z Yeo G Lee F . Single-cell and bulk transcriptome sequencing identifies two epithelial tumor cell states and refines the consensus molecular classification of colorectal cancer. Nat Genet. (2022) 54:963–75. doi: 10.1038/s41588-022-01100-4 35773407 PMC9279158

[7] Lee HO Hong Y Etlioglu HE Cho YB Pomella V Van den Bosch B . Lineage-dependent gene expression programs influence the immune landscape of colorectal cancer. Nat Genet. (2020) 52:594–603. doi: 10.1038/s41588-020-0636-z 32451460

[8] Uhlitz F Bischoff P Peidli S Sieber A Trinks A Lüthen M . Mitogen-activated protein kinase activity drives cell trajectories in colorectal cancer. EMBO Mol Med. (2021) 13:e14123. doi: 10.15252/emmm.202114123 34409732 PMC8495451

[9] Pelka K Hofree M Chen JH Sarkizova S Pirl JD Jorgji V . Spatially organized multicellular immune hubs in human colorectal cancer. Cell. (2021) 184:4734–4752.e20. doi: 10.1016/j.cell.2021.08.003 34450029 PMC8772395

[10] Khaliq AM Erdogan C Kurt Z Turgut SS Grunvald MW Rand T . Refining colorectal cancer classification and clinical stratification through a single-cell atlas. Genome Biol. (2022) 23:113. doi: 10.1186/s13059-022-02677-z 35538548 PMC9092724

[11] Liu Z Hu Y Xie H Chen K Wen L Fu W . Single-cell chromatin accessibility analysis reveals the epigenetic basis and signature transcription factors for the molecular subtypes of colorectal cancers. Cancer Discov. (2024) 14:1082–105. doi: 10.1158/2159-8290.CD-23-1445 38445965

[12] Kotliar D Veres A Nagy MA Tabrizi S Hodis E Melton DA . Identifying gene expression programs of cell-type identity and cellular activity with single-cell RNA-seq. Elife. (2019) 8:e43803. doi: 10.7554/eLife.43803 31282856 PMC6639075

[13] Aibar S González-Blas CB Moerman T Huynh-Thu VA Imrichova H Hulselmans G . SCENIC: single-cell regulatory network inference and clustering. Nat Methods. (2017) 14:1083–6. doi: 10.1038/nmeth.4463 28991892 PMC5937676

[14] Van de Sande B Flerin C Davie K De Waegeneer M Hulselmans G Aibar S . A scalable SCENIC workflow for single-cell gene regulatory network analysis. Nat Protoc. (2020) 15:2247–76. doi: 10.1038/s41596-020-0336-2 32561888

[15] Jiang J Chen S Tsou T McGinnis CS Khazaei T Zhu Q . D-SPIN constructs regulatory network models from scRNA-seq that reveal organizing principles of perturbation response. Cell. (2026) 189:4159–4184.e35. doi: 10.1016/j.cell.2026.04.028 42127893 PMC13387596

[16] Staub E Groene J Heinze M Mennerich D Roepcke S Klaman I . An expression module of WIPF1-coexpressed genes identifies patients with favorable prognosis in three tumor types. J Mol Med (Berl). (2009) 87:633–44. doi: 10.1007/s00109-009-0467-y 19399471 PMC2688022

[17] Kirzin S Marisa L Guimbaud R de Reyniès A Legrain M Laurent-Puig P . Sporadic early-onset colorectal cancer is a specific sub-type of cancer: a morphological, molecular and genetics study. PLoS One. (2014) 9:e103159. doi: 10.1371/journal.pone.0103159 25083765 PMC4118858

[18] Marisa L de Reyniès A Duval A Selves J Gaub MP Vescovo L . Gene expression classification of colon cancer into molecular subtypes: characterization, validation, and prognostic value. PLoS Med. (2013) 10:e1001453. doi: 10.1371/journal.pmed.1001453 23700391 PMC3660251

[19] Smith JJ Deane NG Wu F Merchant NB Zhang B Jiang A . Experimentally derived metastasis gene expression profile predicts recurrence and death in patients with colon cancer. Gastroenterology. (2010) 138:958–68. doi: 10.1053/j.gastro.2009.11.005 19914252 PMC3388775

[20] Wolock SL Lopez R Klein AM . Scrublet: computational identification of cell doublets in single-cell transcriptomic data. Cell Syst. (2019) 8:281–291.e9. doi: 10.1016/j.cels.2018.11.005 30954476 PMC6625319

[21] Bernstein NJ Fong NL Lam I Roy MA Hendrickson DG Kelley DR . Solo: doublet identification in single-cell RNA-seq via semi-supervised deep learning. Cell Syst. (2020) 11:95–101.e5. doi: 10.1016/j.cels.2020.05.010 32592658

[22] Lopez R Regier J Cole MB Jordan MI Yosef N . Deep generative modeling for single-cell transcriptomics. Nat Methods. (2018) 15:1053–8. doi: 10.1038/s41592-018-0229-2 30504886 PMC6289068

[23] Patel AP Tirosh I Trombetta JJ Shalek AK Gillespie SM Wakimoto H . Single-cell RNA-seq highlights intratumoral heterogeneity in primary glioblastoma. Science. (2014) 344:1396–401. doi: 10.1126/science.1254257 24925914 PMC4123637

[24] Yu Q Li YY Chen Y . scMalignantFinder distinguishes Malignant cells in single-cell and spatial transcriptomics by leveraging cancer signatures. Commun Biol. (2025) 8:504. doi: 10.1038/s42003-025-07942-y 40148533 PMC11950360

[25] Subramanian A Tamayo P Mootha VK Mukherjee S Ebert BL Gillette MA . Gene set enrichment analysis: a knowledge-based approach for interpreting genome-wide expression profiles. Proc Natl Acad Sci USA. (2005) 102:15545–50. doi: 10.1073/pnas.0506580102 16199517 PMC1239896

[26] Liberzon A Birger C Thorvaldsdóttir H Ghandi M Mesirov JP Tamayo P . The Molecular Signatures Database (MSigDB) hallmark gene set collection. Cell Syst. (2015) 1:417–25. doi: 10.1016/j.cels.2015.12.004 26771021 PMC4707969

[27] Kang M Gulati GS Brown EL Qi Z Avagyan S Almagro Armenteros JJ . Improved reconstruction of single-cell developmental potential with CytoTRACE 2. Nat Methods. (2025) 22:2258–63. doi: 10.1038/s41592-025-02857-2 41145665 PMC12615260

[28] Hänzelmann S Castelo R Guinney J . GSVA: gene set variation analysis for microarray and RNA-seq data. BMC Bioinf. (2013) 14:7. doi: 10.1186/1471-2105-14-7 23323831 PMC3618321

[29] Becht E Giraldo NA Lacroix L Buttard B Elarouci N Petitprez F . Estimating the population abundance of tissue-infiltrating immune and stromal cell populations using gene expression. Genome Biol. (2016) 17:218. doi: 10.1186/s13059-016-1070-5 27765066 PMC5073889

[30] Eide PW Bruun J Lothe RA Sveen A . CMScaller: an R package for consensus molecular subtyping of colorectal cancer pre-clinical models. Sci Rep. (2017) 7:16618. doi: 10.1038/s41598-017-16747-x 29192179 PMC5709354

[31] Friedman J Hastie T Tibshirani R . Regularization paths for generalized linear models via coordinate descent. J Stat Softw. (2010) 33:1–22. doi: 10.18637/jss.v033.i01 20808728 PMC2929880

[32] Ishwaran H Kogalur UB Blackstone EH Lauer MS . Random survival forests. Ann Appl Stat. (2008) 2:841–60. doi: 10.1214/08-AOAS169 42604595

[33] Chen T Guestrin C . (2016). “ XGBoost: a scalable tree boosting system”, in: Proceedings of the 22nd ACM SIGKDD International Conference on Knowledge Discovery and Data Mining (San Francisco, CA: Association for Computing Machinery), 785–94. doi: 10.1145/2939672.2939785

[34] Lundberg SM Erion G Chen H DeGrave A Prutkin JM Nair B . From local explanations to global understanding with explainable AI for trees. Nat Mach Intell. (2020) 2:56–67. doi: 10.1038/s42256-019-0138-9 32607472 PMC7326367

[35] Binder H Schumacher M . Allowing for mandatory covariates in boosting estimation of sparse high-dimensional survival models. BMC Bioinf. (2008) 9:14. doi: 10.1186/1471-2105-9-14 18186927 PMC2245904

[36] Wolf FA Angerer P Theis FJ . SCANPY: large-scale single-cell gene expression data analysis. Genome Biol. (2018) 19:15. doi: 10.1186/s13059-017-1382-0 29409532 PMC5802054

[37] Tanjak P Chaiboonchoe A Suwatthanarak T Thanormjit K Acharayothin O Chanthercrob J . Tumor-immune hybrid cells evade the immune response and potentiate colorectal cancer metastasis through CTLA4. Clin Exp Med. (2025) 25:2. doi: 10.1007/s10238-024-01515-9 39499374 PMC11538261

[38] Chen Y Wang D Li Y Qi L Si W Bo Y . Spatiotemporal single-cell analysis decodes cellular dynamics underlying different responses to immunotherapy in colorectal cancer. Cancer Cell. (2024) 42:1268–1285.e7. doi: 10.1016/j.ccell.2024.06.009 38981439

[39] Zha B Luo Y Kamili M Zha X . Non-coding RNAs and gastrointestinal cancers prognosis: an umbrella review of systematic reviews and meta-analyses of observational studies. Front Oncol. (2023) 13:1193665. doi: 10.3389/fonc.2023.1193665 37546412 PMC10399243

[40] Gao S Zhao ZY Wu R Zhang Y Zhang ZY . Prognostic value of microRNAs in colorectal cancer: a meta-analysis. Cancer Manag Res. (2018) 10:907–29. doi: 10.2147/CMAR.S157493 29750053 PMC5935085

[41] Li C He X Zhang L Li L Zhao W . A pair-wise meta-analysis highlights circular RNAs as potential biomarkers for colorectal cancer. BMC Cancer. (2019) 19:957. doi: 10.1186/s12885-019-6136-9 31615475 PMC6794748

[42] Inuzuka M Hayakawa M Ingi T . Serinc, an activity-regulated protein family, incorporates serine into membrane lipid synthesis. J Biol Chem. (2005) 280:35776–83. doi: 10.1074/jbc.M505712200 16120614

[43] Zeng Y Xiao D He H He J Pan H Yang W . SERINC2-knockdown inhibits proliferation, migration and invasion in lung adenocarcinoma. Oncol Lett. (2018) 16:5916–22. doi: 10.3892/ol.2018.9403 30405754 PMC6202524

[44] Wang X Jiang C Li Q . Serinc2 drives the progression of cervical cancer through regulating Myc pathway. Cancer Med. (2024) 13:e70296. doi: 10.1002/cam4.70296 39417376 PMC11483714

[45] Sun Y Zhou Y Peng Q Zhou W Li X Wang R . SERINC2-mediated serine metabolism promotes cervical cancer progression and drives T cell exhaustion. Int J Biol Sci. (2025) 21:1361–77. doi: 10.7150/ijbs.105572 39897034 PMC11781177

