# Supplementary material for: Integrated single-cell analysis characterizes malignant epithelial programs and context-dependent immune associations of MP7/SERINC2 in colorectal cancer
Source: Front Immunol. 2026 Sep 3;17:1918381. doi: 10.3389/fimmu.2026.1918381 (PMC13582626; doi:10.3389/fimmu.2026.1918381)
Supplement: Supplementary file 1 [file Table1.docx]

**Supplementary Methods**

**SM1. Sensitivity of scVI integration to the batch covariate**

Batch-key sensitivity was evaluated using a fixed subset of 79,518 cells drawn from all 199 samples. Rare cell types were supplemented during sampling so that all 25 atlas annotations were represented. The counts layer contained nonnegative integer UMI counts. Three scVI models used the same cells, 4,000 genes, random seed, architecture, batch size, and 120 training epochs; only the batch covariate differed (sample_uid, GEO dataset, or no batch covariate). Dataset mixing was quantified by graph mixing and the reversed dataset silhouette width. Preservation of biological structure was evaluated by cell-type neighborhood purity, agreement between Leiden clusters and cell-type labels, leave-one-dataset-out cell-type classification, patient-grouped classification of tumor versus normal epithelium, and distances between paired tumor and normal epithelial centroids.

**SM2. Patient-aware assessment of malignant epithelial cells**

For each epithelial cell, CNV burden was calculated as the mean absolute amplitude of the gene-ordered, smoothed inferCNV profile. A patient-specific CNV-high threshold was defined as the 97.5th percentile of normal epithelial cells from the same patient when at least 20 reference cells were available (44 of 85 patients). For the remaining 41 patients, the 97.5th percentile of normal epithelial cells from the same GEO dataset was used. CNV-high status was treated as supporting evidence because transcriptome-derived CNV profiles may not identify near-diploid malignant cells.

The pretrained pan-cancer logistic model in scMalignantFinder v1.2.0 was applied after normalizing raw counts to 10,000 counts per cell. Of 2,707 model features, 2,639 were present and 68 absent features were set to zero. Classifier support was assigned when the predicted malignancy probability exceeded the 97.5th percentile among normal epithelial cells from the same GEO dataset. The classifier-supported set was restricted to tumor-derived cells in the primary 22,848-cell population. Because GSE132465 and GSE144735 contributed to the published development of scMalignantFinder, this analysis was used as a method-based sensitivity assessment.

A second conservative set used a leave-one-dataset-out expression classifier fitted to the selected scVI latent representation. Positive training labels were tumor-derived cells exceeding the same-dataset normal 99th percentile for both immune-reference and epithelial-reference CNV burdens; negative labels were normal epithelial cells below the corresponding 95th percentiles. A class-balanced logistic regression with standardized features was trained on four datasets and applied to the held-out dataset. Expression support required a probability above the held-out dataset's normal-epithelial 97.5th percentile. The conservative definition required tumor origin, expression support, and either both CNV burdens above their same-dataset normal 97.5th percentiles or membership in a supported CNV clone. Supported clones contained at least 50 tumor cells, no more than 5% normal cells, and at least 10 concordant CNV-high anchors, with an anchor-enrichment odds ratio of at least 2 and Benjamini-Hochberg FDR below 0.05 by one-sided Fisher's exact test. The five-state compositions were compared by total variation distance, and the association between SERINC2 expression and previously estimated MP7 usage was compared by Spearman correlation. cNMF was not refitted after filtering.

**SM3. Recurrence of malignant epithelial states and cell-cycle sensitivity**

The five state labels were evaluated without reclustering or relabeling the primary malignant epithelial-cell population. State-specific counts and within-patient fractions were calculated for each patient, sample, and dataset. Robust detection required at least 10 cells and at least 1% of a patient's malignant epithelial cells. The top 20 markers for each state were converted to standardized expression scores and summarized for patient-state groups containing at least 10 cells. Marker specificity was tested with linear mixed-effects models containing matching-state status and dataset as fixed effects and patient as a random intercept. For leave-one-dataset-out classification, training markers were selected from patient-state pseudobulks in four datasets, and a class-balanced multinomial logistic model was fitted after limiting each patient-state group to 100 cells. Balanced accuracy and macro-F1 were calculated in the held-out dataset at cell and pseudobulk levels.

Cell-cycle sensitivity was assessed by removing 94 detected canonical S- and G2/M-phase genes and rerunning CytoTRACE2 with the same annotations, random seed, and computational settings. Original and cycle-depleted scores were compared by Spearman correlation. Within-patient TUBA1B-positive contrasts required at least 10 TUBA1B-positive cells and 10 cells from the other states. Sample-state summaries containing at least 10 cells were analyzed using patient-fixed-effect linear models with patient-clustered robust standard errors, before and after adjustment for median S- and G2/M-phase scores.

**SM4. Cross-dataset recurrence of pySCENIC regulons**

GRNBoost2, motif pruning, and AUCell scoring were rerun independently in each dataset containing at least 200 malignant epithelial cells, using integer raw counts, the common 16,126-gene universe, and random seed 20260722. GSE144735 contained 121 eligible cells and was not reconstructed separately. GSE132465 and GSE178341 were capped at 3,000 cells by patient-by-state proportional sampling; GSE166555 and GSE200997 contributed 2,755 and 824 cells, respectively. A regulon met the network-recurrence criterion when it was present and its target-gene overlap with the pooled regulon had a Benjamini-Hochberg-adjusted hypergeometric FDR below 0.05 in at least two datasets. Within each patient, state-specific activity was calculated as the mean AUCell z score in the target state minus the mean in the other states, requiring at least five target-state and 20 comparison cells. Activity recurrence required at least five eligible patients, a positive effect in at least 70% of patients, a positive dataset median in at least two datasets, and a one-sided Wilcoxon FDR below 0.05. Regulons satisfying both network and activity criteria were classified as recurrent. AUCell scores were also modeled with patient fixed effects, patient-clustered standard errors, and within-patient-centered CNV score.

**SM5. Within-patient cNMF and epithelial-lineage comparisons of MP7**

Tumors containing at least 200 malignant epithelial cells were factorized independently from integer raw counts. The analysis included 26 patients and 21,209 cells from four datasets. For each patient, ranks K=4, 6, and 8 were fitted with 30 starts, 2,000 patient-specific highly variable genes, a maximum of 800 iterations, density threshold 0.5, local-neighborhood size 0.3, and random seed 20260723. Patient-level programs were matched to the pooled MP1-MP8 loadings using Spearman correlation and top-100 gene overlap. A high-confidence match required rho at least 0.20, at least 10 shared top-100 genes, and a Benjamini-Hochberg-adjusted hypergeometric FDR below 0.05. Reciprocal-best matches were also recorded.

The MP7 top-100 genes were scored in 117,447 annotated epithelial cells after normalization to 10,000 counts per cell and log transformation. The weighted top-50 score used nonnegative cNMF spectrum weights normalized to sum to one. Comparisons used sample-level means from epithelial groups containing at least 10 cells and rank-based linear models with dataset fixed effects and patient-clustered standard errors. Secretory-lineage similarity was calculated by Spearman correlation between sample-median MP7 top-100 expression profiles in normal goblet and MP7-dominant malignant cells. For transfer to TCGA, a 15-gene malignant-lineage signature was derived from the MP7 top-100 genes using malignant-state and goblet-lineage contrasts without using TCGA expression or survival outcomes. The MP7 top-100 and 15-gene lineage scores were compared with malignant-cell pseudobulk MP7 usage, TCGA tumor purity, normal goblet-lineage expression, MCP-counter immune and stromal estimates, and continuous Cox models.

**SM6. Survival-model and external-cohort analyses**

Optimism in the complete MP7-derived modeling pipeline was assessed by 20 repeats of five-fold nested cross-validation. Within each outer training set, univariable screening of the 298 detected MP7 genes and inner five-fold LASSO-Cox fitting were repeated without access to the outer test fold. In a separate 20 by five-fold analysis, the fixed 17-gene model, a clinical model containing age, sex, and simplified pathological stage, and their combination were compared by held-out Harrell C-index, calibration slope, and inverse-probability-of-censoring-weighted Brier scores at 3 and 5 years.

The TCGA-derived coefficients were also evaluated in GSE39582 and GSE17536. UGT2B7 and FAM177B were absent from the GPL570 platform, so these analyses used the 15 available genes. Multiple probes for each gene were standardized across samples and summarized by the sample-wise median. External gene scores were converted to within-cohort mid-rank probabilities and mapped gene by gene to the corresponding empirical TCGA quantiles using R quantile type 8 before application of the TCGA coefficients. No outcome information was used in probe summarization, mapping, or coefficient application. For SERINC2, all four GPL570 probes were standardized and summarized by their patient-level median. Continuous SERINC2 expression was tested using unadjusted and age-, sex-, and stage-adjusted Cox models, followed by fixed-effect meta-analysis.

**SM7. D-SPIN stability and comparison analyses**

The D-SPIN input combined the primary 22,848 putative malignant epithelial cells with normal-tissue epithelial cells not present in that population. Conditions were defined as sample_uid combined with analysis group (putative malignant or normal epithelium); GEO dataset was the batch variable and normal epithelium was the control. Conditions with fewer than 20 cells were excluded, and at most 400 cells were sampled per condition. SERINC2 was retained as the index gene, and the remaining candidates were selected from a dataset-aware Seurat-v3 highly variable-gene ranking after excluding mitochondrial, ribosomal, hemoglobin, immunoglobulin, and selected technical genes. The main model requested SERINC2 plus 159 highly variable genes; 158 genes remained after filtering. The undirected pseudo-likelihood model used 25,810 cells from 106 conditions (42 malignant and 64 normal), 240 epochs, and a filtering threshold of 0.003.

Robustness was assessed in 15 refits comprising two alternative optimization seeds, four 80% condition subsamples, five leave-one-dataset-out fits, one independent discretization, and three feature-set sizes (120, 200, and 300 requested genes). A stable SERINC2 edge was defined as a partner ranked among the 40 strongest main-model SERINC2 edges and recovered among the top 20 edges with the same sign in at least 80% of eligible refits. The conditional null comprised 30 models in which the discretized SERINC2 state was permuted within each condition; the primary metric was the mean absolute coupling of the 20 strongest SERINC2 edges. For the simpler comparator, condition-level mean expression was rank transformed and residualized for GEO dataset and malignant/control group. Residualized SERINC2 values were correlated with the remaining genes, and significance was assessed by permutations within dataset-by-group blocks with Benjamini-Hochberg correction.

**Supplementary Figure**

**Supplementary Figure S1.** Sensitivity of scVI integration to batch-key definition. All three models were trained on the same 79,518 cells and 4,000 genes using identical architecture and training parameters. (A) UMAPs colored by GEO dataset for sample_uid, dataset, and no-batch settings. (B) Dataset graph-mixing and reversed silhouette scores. (C) Cell-type neighborhood purity, leave-one-dataset-out cell-type balanced accuracy, and patient-grouped tumor-versus-normal epithelial ROC AUC. (D) Paired-patient tumor-normal epithelial centroid distances for 59 patients.

**Supplementary Figure S2.** Patient-aware CNV and expression-classifier assessment of the primary malignant epithelial-cell population. (A) Per-cell CNV-burden distributions for 85 patients relative to the corresponding normal-reference 97.5th percentile. (B) Intersection of patient-aware CNV-high status and dataset-calibrated scMalignantFinder support among 22,848 cells. The 99.0% value includes all classifier-supported cells, whereas the tumor-derived high-confidence population used in panel C contained 22,555 cells (98.7%). (C) Per-patient retention in the tumor-derived high-confidence population. (D) Five-state composition before and after classifier filtering. (E) SERINC2 expression and MP7 usage in the classifier-supported population; correlations for both populations are shown. (F) Retention of 1,068 MP7-dominant cells under the classifier-supported and conservative internal definitions. HC, high confidence.

**Supplementary Figure S3.** Recurrence of the five malignant epithelial states and cell-cycle sensitivity of CytoTRACE2. (A) State-specific cell counts across the five GEO datasets; parentheses indicate patients with any detection and robust detection. (B) Within-patient state fractions for 85 patients. (C) Matching-state effects for the five marker signatures from linear mixed-effects models. Points show estimates and bars show 95% confidence intervals. (D) Leave-one-dataset-out balanced accuracy at patient-state-weighted cell and pseudobulk levels; the dashed line denotes five-class chance. (E) Cell-level agreement between original and cycle-depleted CytoTRACE2 scores. (F) Original and cycle-depleted sample-state distributions. (G) Within-patient TUBA1B-positive contrasts. (H) TUBA1B-positive effects from patient-fixed-effect models before and after adjustment for S- and G2/M-phase scores.

**Supplementary Figure S4.** Cross-dataset and cross-patient recurrence of state-associated pySCENIC regulons. The five regulons with the highest pooled RSS for each state were evaluated. (A) Target-gene Jaccard overlap between pooled and independently reconstructed regulons. Asterisks indicate adjusted hypergeometric FDR below 0.05; x indicates absence of a regulon. (B) Median within-patient AUCell z-score difference between the target state and the other malignant states. Point size represents the fraction of patients with a positive difference. (C) Number of candidates per state meeting the network, patient-activity, or combined criteria.

**Supplementary Figure S5.** Within-patient recovery of pooled cNMF programs. Twenty-six patients with at least 200 malignant epithelial cells were factorized independently at K=4, 6, and 8. (A) Spearman correlation between each pooled MP1-MP8 loading vector and its best patient-level K=8 program. (B) MP7 matching across K=4, 6, and 8. Asterisks indicate a high-confidence match; black boxes indicate reciprocal-best matches. (C) Number of patients with a high-confidence or reciprocal-best match for each pooled program. (D) Recurrence of pooled MP7 top-100 genes among the best qualifying MP7-like program in each matched patient.

**Supplementary Figure S6.** Epithelial-lineage comparison and bulk transfer of MP7. (A) Weighted MP7 signature scores in normal and tumor-associated nonmalignant epithelial groups and in malignant non-MP7 and MP7-dominant cells. Points represent sample-level means. (B) Row-standardized expression of the top 20 MP7 genes in four epithelial groups. (C) Similarity of sample-median MP7 top-100 profiles in normal goblet and MP7-dominant malignant cells. (D) Calibration of the MP7 top-100 and 15-gene lineage scores against mean cNMF MP7 usage in patient-level malignant-cell pseudobulks; colors indicate the source GEO datasets. (E) Correlations of the two TCGA scores with ABSOLUTE purity, a goblet-lineage score, and MCP-counter immune and stromal estimates. (F) Hazard ratios per standard-deviation increase in continuous Cox models with clinical and composition-related covariates. Error bars show 95% confidence intervals.

**Supplementary Figure S7.** Internal and external assessment of the MP7-derived survival model and SERINC2. (A) Held-out C-index after 20 repeats of five-fold nested cross-validation of the complete feature-selection pipeline. (B) Held-out C-index, calibration slope, and 3- and 5-year Brier scores for the clinical, 17-gene, and combined models across 100 test folds. (C) Adjusted hazard ratios per standard-deviation increase in the available 15-gene score in GSE39582 and GSE17536. (D) C-index of the 15-gene, clinical, and combined models in each cohort. UGT2B7 and FAM177B were not available on GPL570. (E) Univariable and age-, sex-, and stage-adjusted associations of continuous SERINC2 expression with overall survival, with fixed-effect meta-analysis. (F) Median and range of pairwise Spearman correlations among the four SERINC2 probes in each cohort.

**Supplementary Figure S8.** RT-qPCR validation of SERINC2 knockdown in SW480 cells. Relative SERINC2 mRNA expression was calculated using the 2^−ΔΔCt^ method, with ACTB as the reference gene and SH-NC as the calibrator. Points represent three independent experiments; bars and error bars indicate the mean and SD. Both shRNAs reduced SERINC2 expression by approximately 94%.

**Supplementary Figure S9.** D-SPIN analysis of the SERINC2 expression context. (A) D-SPIN coupling coefficients for the 17 SERINC2-associated edges meeting the cross-refit stability criterion. (B) Whole-matrix Spearman correlations between the main fit and 15 refits spanning optimization seeds, condition subsampling, leave-one-dataset-out fitting, independent discretization, and feature-set size. (C) Distribution of the mean absolute top-20 SERINC2 coupling across 30 within-condition permutations; the observed value is shown separately. (D) D-SPIN couplings compared with GEO-dataset- and malignant/control-group-adjusted condition-level pseudobulk Spearman associations. Stable edges are highlighted. D-SPIN couplings are undirected statistical dependencies and do not establish direct regulation or causal direction.
